# Unique IL-13Rα2/STAT3 mediated IL-13 regulation detected in lung conventional dendritic cells, 24 h post viral vector vaccination

**DOI:** 10.1038/s41598-020-57815-z

**Published:** 2020-01-23

**Authors:** Sreeja Roy, Ho-Ying Liu, Muhammad Irwan Jaeson, Lachlan Paul Deimel, Charani Ranasinghe

**Affiliations:** Molecular Mucosal Vaccine Immunology Group, Department of Immunology and Infectious Disease, The John Curtin School of Medical Research, The Australian National University, Canberra, ACT 2601 Australia

**Keywords:** Vaccines, Interleukins, Immunology

## Abstract

This study demonstrates that 24 h following viral vector-based vaccination IL-13Rα2 functions as a master sensor on conventional dendritic cells (cDCs), abetted by high protein stability coupled with minimal mRNA expression, to rapidly regulate DC mediated IL-13 responses at the lung mucosae, unlike IL-13Rα1. Under low IL-13, IL-13Rα2 performs as a primary signalling receptor, whilst under high IL-13, acts to sequester IL-13 to maintain homeostasis, both in a STAT3-dependent manner. Likewise, we show that viral vector-derived IL-13 levels at the vaccination site can induce differential STAT3/STAT6 paradigms in lung cDC, that can get regulated collaboratively or independently by TGF-β1 and IFN-γ. Specifically, low IL-13 responses associated with recombinant Fowlpox virus (rFPV) is regulated by early IL-13Rα2, correlated with STAT3/TGF-β1 expression. Whilst, high IL-13 responses, associated with recombinant Modified Vaccinia Ankara (rMVA) is regulated in an IL-13Rα1/STAT6 dependent manner associated with IFN-γR expression bias. Different viral vaccine vectors have previously been shown to induce unique adaptive immune outcomes. Taken together current observations suggest that IL-13Rα2-driven STAT3/STAT6 equilibrium at the cDC level may play an important role in governing the efficacy of vector-based vaccines. These new insights have high potential to be exploited to improve recombinant viral vector-based vaccine design, according to the pathogen of interest and/or therapies against IL-13 associated disease conditions.

## Introduction

IL-13 and IL-4 share a common signalling receptor system and are known to have overlapping as well as distinct functions^1^. These two cytokines have been extensively studied under allergy, asthma, helminth and parasitic infections^2–5^. IL-13 is produced by various immune cell types, specifically innate lymphoid cells (ILC2s), CD4 and CD8 T cells^6,7^ and can directly impact the function of eosinophils, basophils and dendritic cells (DCs)^8,9^. Recent allergy and asthma studies have shown that ILC2-derived IL-13 can stimulate the migration of lung DCs to promote Th2 immunity^10^. Interestingly, whilst overproduction of IL-13 is associated with tissue pathology^11^, deficiency of IL-13 has been associated with increased susceptibility to certain skin cancers^12^. Moreover, mounting evidence has also suggested the importance of IL-13 regulation in infection and immunity.

We have previously demonstrated that the vaccine route, viral vector combination and cytokine milieu (level of IL-13) can significantly alter the adaptive immune outcomes^7,13,14^. Pox viral vector-based HIV vaccine strategies that transiently inhibited IL-13 activity at the vaccination site, can induce high avidity/poly-functional T cells both in mice and macaques^15–17^ (Li *et al*. in preparation). Interestingly, 24 h post delivery of these vaccines, whilst ILC2s were found to be the major source of IL-13 at the vaccination site^18^, elevated recruitment of CD11b^+^ CD103^−^ conventional DCs (cDC) to the lung mucosae were associated with the observed adaptive immune outcomes^19^. Moreover, recently we have shown that different viral vector-based vaccines can induce unique ILC2-derived IL-13 profiles and recruitment of different DC subsets to the vaccination site, 24 h post delivery^20^. Specifically, i.n. rFPV vaccination associated with low ILC2-derived IL-13 recruited CD11b^+^ CD103^−^ conventional DC (cDC)^19^, whilst high/medium ILC2-derived IL-13 producers, rMVA and Adenovirus 5 (Ad5) vaccinations recruited enhanced cross-presenting DCs and plasmacytoid DCs (pDCs) to the lung mucosae, respectively. Using adoptive transfer of different DC subsets to the lung mucosae, we have also shown that cross-presenting DCs induced low avidity HIV-specific T cells, whilst cDC were associated with high avidity T cells^19^.

IL-13 can bind to IL-13Rα1 with low affinity (K_D_ = 30 nM) and, heterodimerize with IL-4Rα subunit to form the Type II IL-4 receptor complex to activate downstream JAK1- or JAK2-/TYK2- induced STAT6 signalling^4^. Cheng *et al*. have also proposed that activation of IL-13Rα1/IL-4Rα could induce STAT3 signalling under certain IL-13 conditions^21^ and a recent study has shown an association of IL-13Rα1 with STAT3 in relation to cardiac homeostasis^22^. Interestingly, IL-13Rα2, known to be the high affinity receptor for IL-13 (K_D_ = 440 pM)^1,23^, initially thought to be a decoy receptor in mice has now been established as a functional receptor in humans^24^. Overexpression of IL-13Rα2 has been associated with various cancers and targeted as an anti-cancer therapeutic^25,26^. Although the exact signalling mechanism of IL-13Rα2 is not yet well-characterised, in malignant glioma, IL-13Rα2 has shown to regulate activation of STAT3^27^ and initiate signalling via activation protein 1 (AP-1). Furthermore IL-13Rα2 has also shown to induce transforming growth factor beta 1 (TGF-β1) under certain chronic infections and autoimmune disease conditions^2^. Recently, we have also shown that in the context of viral vector-based vaccination, the STAT6 independent pathway (likely associated with IL-13Rα2) was involved in antibody differentiation^28^. Therefore, knowing that both STAT3 and STAT6 are involved in IL-13 regulation and that IFN-γ can also modulate IL-13 activity^29–31^, this study focused on deciphering the IL-13 signalling mechanisms lung cDCs employ under different IL-13 conditions (different viral vector-based vaccination conditions), to induce vastly different adaptive immune outcomes.

## Results

### rFPV vaccination significantly up-regulated IL-13Rα2 expression on lung cDCs 24 h post i.n. vaccination

**Figure 1:**
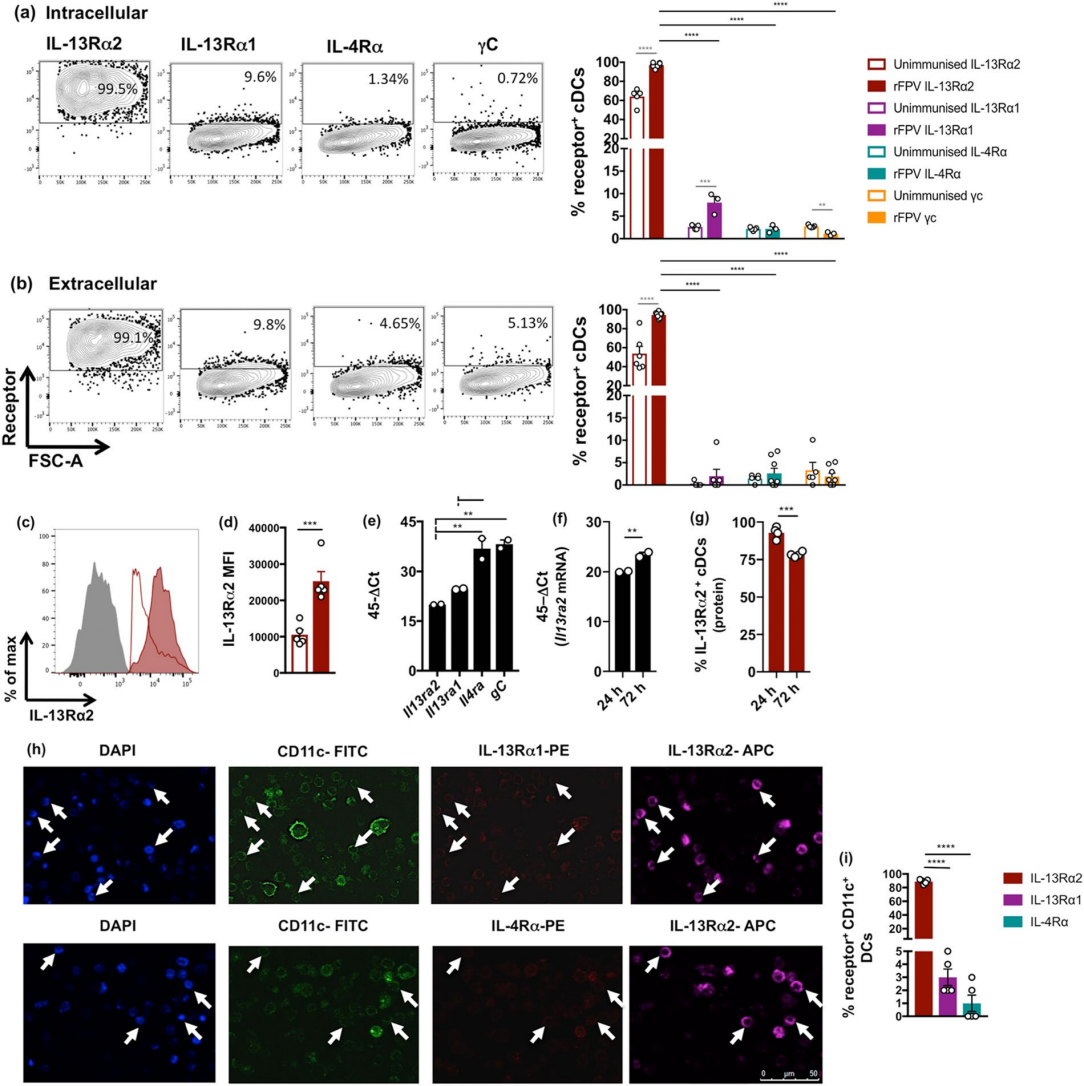
Evaluation of IL-4 and IL-13 receptors on lung cDCs 24 h post rFPV vaccination. BALB/c mice (n = 3–5 per group) were intranasally (i.n.) immunised with rFPV. 24 h post vaccination, lungs were harvested and single cell suspensions were stained for MHC-II^+^ CD11c^+^ CD11b^+^ CD103^−^ cDCs to evaluate receptor expressions on lung cDCs using flow cytometry as described in methods and Fig. S1a–d. Representative flow cytometry plots (left panel) and bar graphs (right panel) show the percentage of cDCs expressing IL-13Rα2, IL-13Rα1, IL-4Rα and γc at the **(a)** intracellular and **(b)** extracellular levels compared between rFPV vaccinated and unimmunised mice. **(c)** FACS histogram plot and **(d)** bar graph show a comparative extracellular IL-13Rα2 expression density on lung cDCs from rFPV vaccinated (solid red) compared to unimmunised mice (red line) and isotype control (solid grey). In all graphs, error bars represent Standard Error of mean (SEM) and *p* values were calculated using One-way ANOVA followed by Tukey’s multiple comparison test (black), and paired Student’s t-test (grey). Bar graphs show mRNA expression level of **(e)** *Il13ra2*, *Il13ra1*, *Il4ra* and *gC* at 24 h and **(f)** *Il13ra2* at 24 and 72 h post rFPV vaccination, in 500 sorted lung cDCs, evaluated using qPCR, represented as 45-ΔCt, as described in materials and methods. **(g)** Bar graph represents percentage of lung cDCs expressing IL-13Rα2 at 24 and 72 h post rFPV vaccination measured using flow cytometry as described in methods. Error bars represent Standard Error of mean (SEM) and *p* values were calculated using One-way ANOVA followed by Tukey’s multiple comparison test. *p < 0.05, **p < 0.01, ***p < 0.001, ****p < 0.0001. **(h)** Representative immunofluorescence images show lung cells from i.n. rFPV vaccinated BALB/c mice (n = 5) 24 h post-delivery, expressing IL-13Rα2^+^ and IL-13Rα1^+^ (top panel) and IL-13Rα2^+^ and IL-4Rα^+^ (bottom panel) at magnification x60, as described in methods and Fig. S2a. White arrows indicate CD11c^+^ DCs either IL-13Rα2^+^ IL-13Rα1^+^ or IL-13Rα2^+^ IL-4Rα^+^. **(i)** Bar graph shows the significant differences between percentage of cDCs expressing IL-13Rα2^+^, IL-13Rα1^+^, or IL-4Rα^+^. All experiments were repeated three times, except mRNA studies, which were repeated twice.

Knowing that rFPV priming, which induced low ILC2-derived IL-13 and CD11b^+^ CD103^−^ cDCs^20^, was associated with high avidity T cells^19^, this study aimed to unravel the underlying mechanisms by which IL-13 regulated cDC recruitment, following intranasal (i.n.) rFPV vaccination. Hence, IL-4/IL-13 receptor expression on lung cDCs (MHC-II^+^ CD11c^+^ CD11b^+^ CD103^−^) were evaluated 24 h post delivery using flow cytometry. Data revealed that infiltrated lung cDCs in response to 24 h of i.n. rFPV vaccination exhibited significantly higher proportion of intracellular and extracellular expression of IL-13Rα2 compared to the unimmunised control (*p* < 0.0001; Fig. 1a,b). In the context of other IL-4/IL-13 associated receptors, IL-4Rα, IL-13Rα1 and γc were marginally or not expressed on cDCs **(***p* < 0.001; Fig. 1a,b). Upon vaccination although intracellular IL-13Rα1 expression was up-regulated compared to the unimmunised control (*p* = 0.0019), no such difference was observed extracellularly **(**Fig. 1a,b**)**. Moreover, unlike the other receptors, significantly higher IL-13Rα2 density was also observed on vaccinated lung cDCs compared to the unimmunized control (*p* = 0.0006) **(**Fig. 1c,d**)**. Note that to validate the specificity of IL-4/IL-13 receptor antibodies, expression of these receptors was assessed on several different immune cells as well as tissue types. Interestingly, elevated IL-13Rα2 expression was only observed on vaccinated lung DCs not splenic (systemic) DCs or other immune cells (CD4^+^ T cells, CD8^+^ T cells and B220^+^ B cells) tested from both tissue types (Fig. S1c,d), indicating that the IL-13Rα2 expression pattern was lung DC-specific.

Interestingly, qPCR analysis of IL-4/IL-13 mRNA expression on lung cDCs at 24 h post rFPV vaccination revealed that *Il13ra2* mRNA expression was significantly lower (associated with high Ct) (Figs. 1e, S1e) compared to all the other receptors, where *Il4ra* and *gC* mRNA expression levels were much greater than *Il13ra1* and *Il13ra2* (*Il13ra2* vs *Il4ra p* = 0.0034, *Il13ra2* vs *gC p* = 0.0018), (Fig. 1e). However, in the context of IL-13Rα2, at 72 h post rFPV vaccination, elevated mRNA followed by reduced protein expression was observed (inverse to 24 h) (Fig. 1f,g), indicative of a non-linear mRNA-protein regulation of this receptor.

To further confirm the expression profiles of IL-13Rα2, IL-13Rα1 and IL-4Rα 24 h post rFPV vaccination on lung DCs, immunofluorescence staining was also performed as described in methods and Fig. S2a. Data showed that elevated proportion of lung CD11c^+^ DCs expressed IL-13Rα2, compared to IL-13Rα1 or IL-4Rα, (*p* < 0.0001) in accordance with flow cytomtery data **(**Fig. 1h,i).

### IL-13 stimulation conditions lead to differential expression of IL-13Rα1 and IL-13Rα2 on CD11c^+^ lung DCs

**Figure 2:**
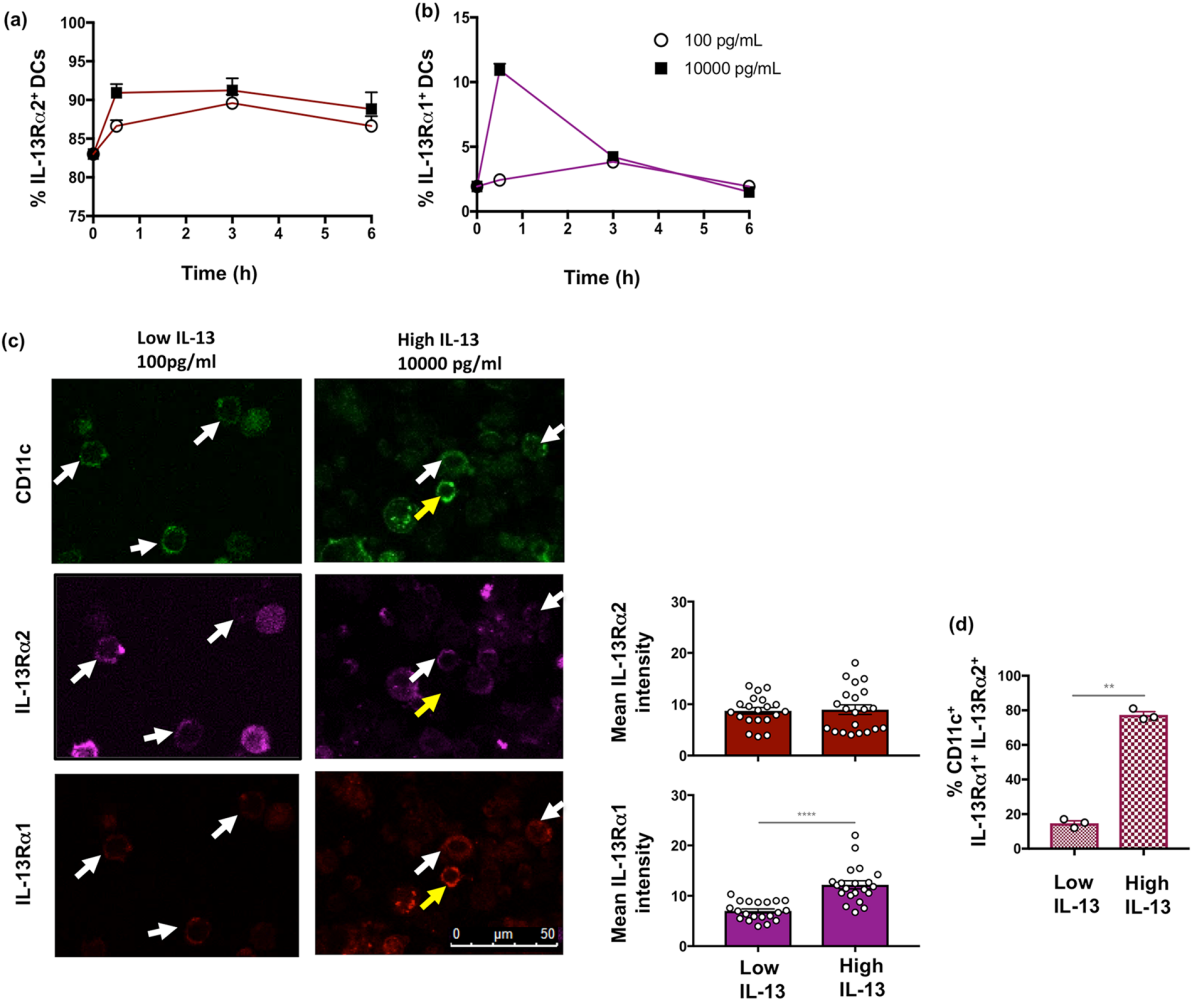
Evaluation of relative IL-13Rα1 and IL-13Rα2 expression on lung DCs following low and high IL-13 stimulation *in vitro*. Lung cells from unimmunised BALB/c (n = 5 per group) were stimulated with 100 (low) or 10000 pg/ml (high) IL-13 for 0.5, 3 and 6 h. Lung suspensions were stained for MHC-II^+^ CD11c^+^ DCs and IL-13 receptor expression was evaluated using flow cytometry. Line graphs show percentage of lung cDCs expressing **(a)** IL-13Rα2 and **(b)** IL-13Rα1 in response to low or high IL-13 concentrations over time. Lung suspensions stained with anti-mouse CD11c-FITC, IL-13Rα1 and IL-13Rα2 were also used for confocal microscopy as described in methods and Fig. S2b,c. **(c)** Representative images at magnification x60 show corresponding CD11c (top), IL-13Rα2 (middle) and IL-13Rα1 (bottom) expression as well as quantified mean intensity for IL-13Rα2 (red bars) and IL-13Rα1 (magenta bars) at 10000 pg/ml (high) and 100 pg/ml (low) IL-13 conditions, stimulated for 0.5 h, as described in methods. White arrows indicate CD11c^+^ DCs co-expressing IL-13Rα2 and IL-13Rα1, whilst yellow arrows show expression of IL-13Rα1 only. **(d)** Bar graph indicates the percentage of CD11c^+^ DCs co-expressing IL-13Rα2 and IL-13Rα1 under low and high IL-13 stimulation conditions. Error bars represent Standard Error of mean (SEM) and *p* values were calculated using paired Student’s t-test. *p < 0.05, **p < 0.01, ***p < 0.001, ****p < 0.0001 and these experiments were repeated three times.

As different viral vector-based vaccines have shown to induce different levels of IL-13 at the lung mucosae, which influence DC activity^20^, *in vitro* IL-13 stimulation was performed to mimic these vaccination conditions in order to study the effect of IL-13 on IL-4/IL-13 receptors. Flow cytometric analysis showed that when unimmunized lung cells from BALB/c mice were stimulated with a range of IL-13 concentrations, at different time intervals, IL-13Rα1 and IL-13Rα2 were differentially expressed. Within 30 minutes of low IL-13 (100 pg/ml) stimulation, IL-13Rα2 was expressed, and was sustained even at 10000 pg/ml (10 ng/ml) IL-13 concentration **(**Fig. 2a). In contrast, only very high IL-13 concentrations, 10000 pg/ml (10 ng/ml) lead to the expression of IL-13Rα1 and the expression was time dependent, where at 6 h the expression level was similar to the baseline control, unlike IL-13Rα2 **(**Fig. 2b). Confocal imaging as described in methods and Figs. S2b,c and S3 further confirmed that very high IL-13 10000 pg/ml (10 ng/ml) can induce elevated expression of IL-13Rα1 on lung CD11c^+^ DCs compared to no or low IL-13 (100 pg/ml) conditions (*p* < 0.0001) **(**Fig. 2c top and bottom panels**)**. In contrast, both high and low IL-13 conditions, showed no difference in IL-13Rα2 expression on lung CD11c^+^ DCs, consistent with flow cytometry **(**Fig. 2c top and middle panels**)**. Moreover, an average 77% and 15% of lung CD11c^+^ DCs were found to co-express IL-13Rα2 and IL-13Rα1 under high and low IL-13 conditions respectively (Fig. 2d). Confocal microscopy also further confirmed that there was no IL-4Rα activity following IL-13 stimulation (data not shown).

### STAT3 inhibition significantly up-regulated IL-13Rα2 and down-regulated IL-13Rα1 on lung DCs

**Figure 3:**
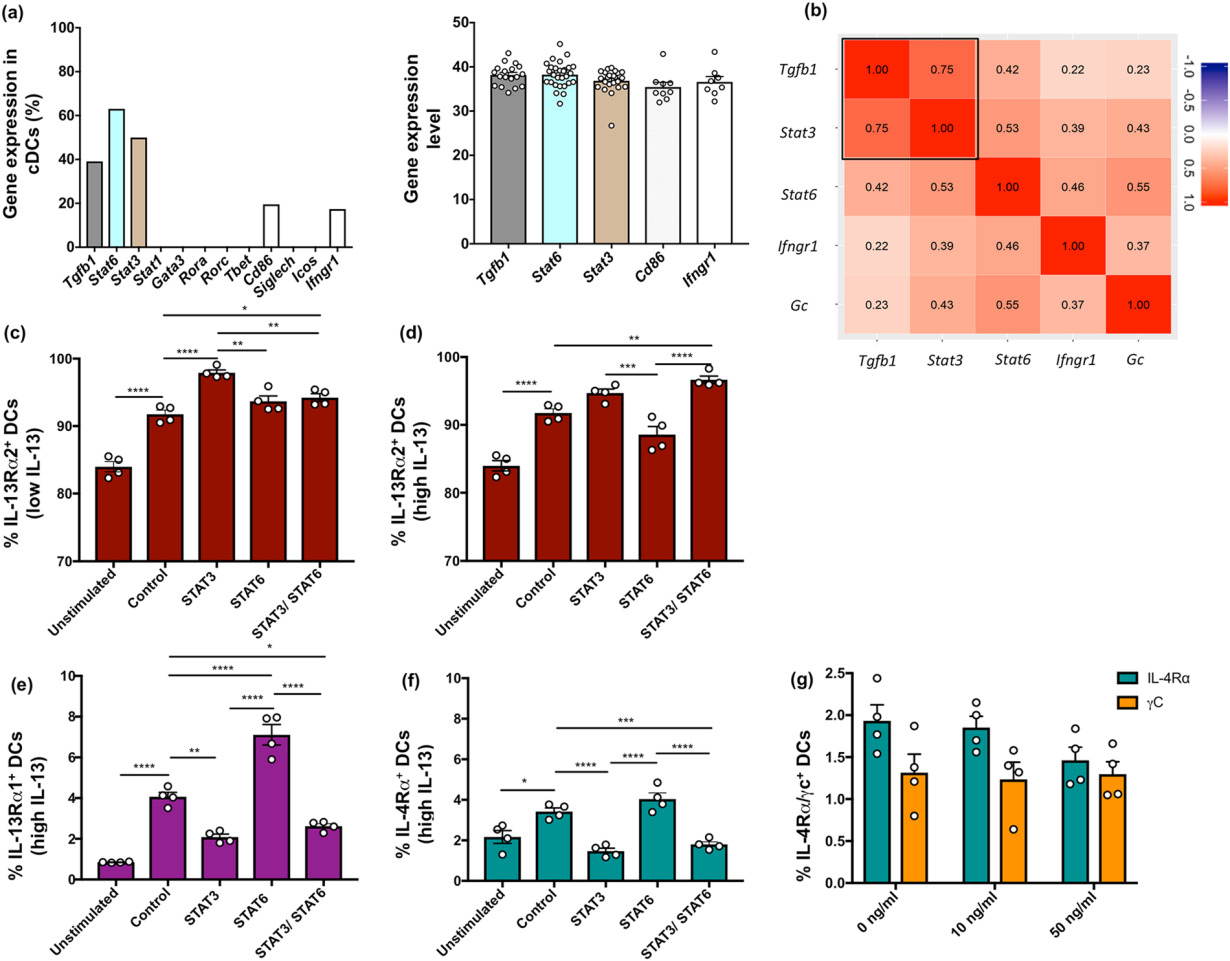
Expression of IL-4/IL-13 receptors and related molecules following vaccination and *in vitro* IL-13 stimulation. BALB/c mice (n = 3 per group) were vaccinated i.n. with rFPV and MHC-II^+^ CD11c^+^ CD11b^+^ CD103^−^ single cDCs were sorted for Fluidigm 48.48 Biomark assay to analyse the expression of 12 selected genes as described in methods. **(a)** Graphs represent the percentage of cDCs expressing the genes of interest (left) and the expression level for each gene represented as 40^−ΔCt^ (where 40 represent the maximum number of qPCR cycles) (right). **(b)** Principal Component Analysis (PC1 vs PC2) was performed on the genes of interest as described in methods. Correlation data indicate the level of expression where values closest to 1.00 represent the strongest correlation. **(c)** Graphs indicate expression of IL-13Rα2 on lung MHC-II^+^ CD11c^+^ DCs from BALB/c mice (n = 4) following STAT3, STAT6 or combined STAT3/STAT6 inhibition under no stimulation (unstimulated), 100 pg/ml (low) and **(d)** 10000 pg/ml (high) IL-13 concentrations for 3 h, *in vitro*. **(e**,**f)** IL-13Rα1 and IL-4Rα receptor expression under the same conditions and **(g)** IL-4Rα and γC expression on unimmunised lung DCs following 0, 10 and 50 ng/ml of IL-4 stimulation for 0.5 h. Error bars represent Standard Error of mean (SEM) and *p* values were calculated using One-way ANOVA followed by Tukey’s multiple comparison test. *p < 0.05, **p < 0.01, ***p < 0.001, ****p < 0.0001. Experiments were repeated two to three times.

IL-13Rα1 signalling is known to activate STAT6^1^, and in some cases STAT3^22,30^, and IL-13Rα2 has shown to activate STAT3 and TGF-β1^2,27^. Furthermore, our recent studies have shown *Stat3*, *Stat6* and *Tgfb1* gene expression on lung ILC2s, 24 h following viral vector vaccination (Jaeson *et al*. submitted). Knowing that ILC2-derived cytokines, especially IL-13, can impact DC recruitment^20^, in this study, 12 regulatory genes were assessed by single cell Fluidigm 48.48 assay as described in materials and methods and Fig. S4. Data revealed that, 40–60% of cDCs expressed *Tgfb1, Stat3* and *Stat6*, 24 h post rFPV vaccination **(**Fig. 3a**)**. Also, 15–20% of cDCs were found to express *Ifngr1* and *cd86*. The *cd86* expression as opposed to *siglec-h* further confirmed that the sorted single cells were cDCs and not pDCs (Fig. 3a). Principal component Analysis (PCA) revealed that, the probability of co-expression of *Stat3* and *Tgfb1* on cDCs was much greater (75%) than *Tgfb1* and *Stat6* (42%) (Fig. 3b), and co-expression of *Stat3* together with *Stat6* was (53%), 24 h post rFPV vaccination (Fig. 3b). Furthermore, the probability of co-expression of *Ifngr1* with *Stat3* whilst being 39%, *Ifngr1 with Tgfb1* was 22%, which were much lower than co-expression of *Ifngr1* and *Stat6* (46%) **(**Fig. 3b**)**. Note that in these studies, Ribosomal protein L32 (*Rpl32*), Stratifin (*Ywhas*) and Eukaryote elongation factor 2 (*Eef2*) were used as endogenous positive control genes to validate the mRNA data **(**Table S1**)**.

To understand the relationship between STAT3, STAT6 and IL-13Rα2 at the protein level (by mimicking low and high IL-13 conditions at the vaccination site post different viral vector-based vaccination), when lung cells were treated with small-molecule inhibitors of STAT3 or STAT6 in the presence of low (100 pg/ml) and high (10000 pg/ml or 10 ng/ml) IL-13, differential regulation of IL-13Rα2 was detected on lung DCs. These results clearly demonstrated that under low IL-13 stimulatory conditions, STAT3 inhibition caused significant up-regulation of IL-13Rα2 compared to the uninhibited control (*p* < 0.001) **(**Fig. 3c, S5a**)**. In contrast, under these conditions, although STAT6 inhibition showed some up-regulation of IL-13Rα2, combined STAT3/STAT6 inhibition did not show any change in IL-13Rα2 expression compared to STAT6 inhibition alone, although there was some up-regulation compared to the control (*p* = 0.026) **(**Figs. 3c, S5a). But surprisingly, under high IL-13, both STAT3 inhibition and combined STAT3/STAT6 inhibition induced elevated IL-13Rα2 expression on DCs (Figs. 3d, S5b). Under all inhibitory conditions tested, the profiles of IL-13Rα1 and IL-4Rα expression mimicked each other (Figs. 3e,f, S5c,d). Specifically, STAT6 inhibition caused significant up-regulation of these two receptors on DCs compared to the uninhibited control. In contrast, STAT3 and combined STAT3/STAT6 inhibition showed a significant down-regulation of IL-13Rα1 and IL-4Rα compared to the uninhibited control (Figs. 3e,f, S5c,d). Note that, STAT6 inhibition induced IL-13Rα1 up-regulation, further confirming the association of IL-13Rα1 with STAT6. Therefore, following STAT3 inhibition up-regulation of IL-13Rα2 was indicative of the IL-13Rα2 association with STAT3. It is also noteworthy that, IL-4 receptors (IL-4Rα and γc) were not regulated on DCs even upon IL-4 stimulation **(**Figs. 3g, S6). This confirmed that the observed receptor regulation was triggered by IL-13 not IL-4.

### STAT3 inhibition significantly down-regulated TGF-β1 on lung cDCs *in vivo*, associated with IL-13Rα2

**Figure 4:**
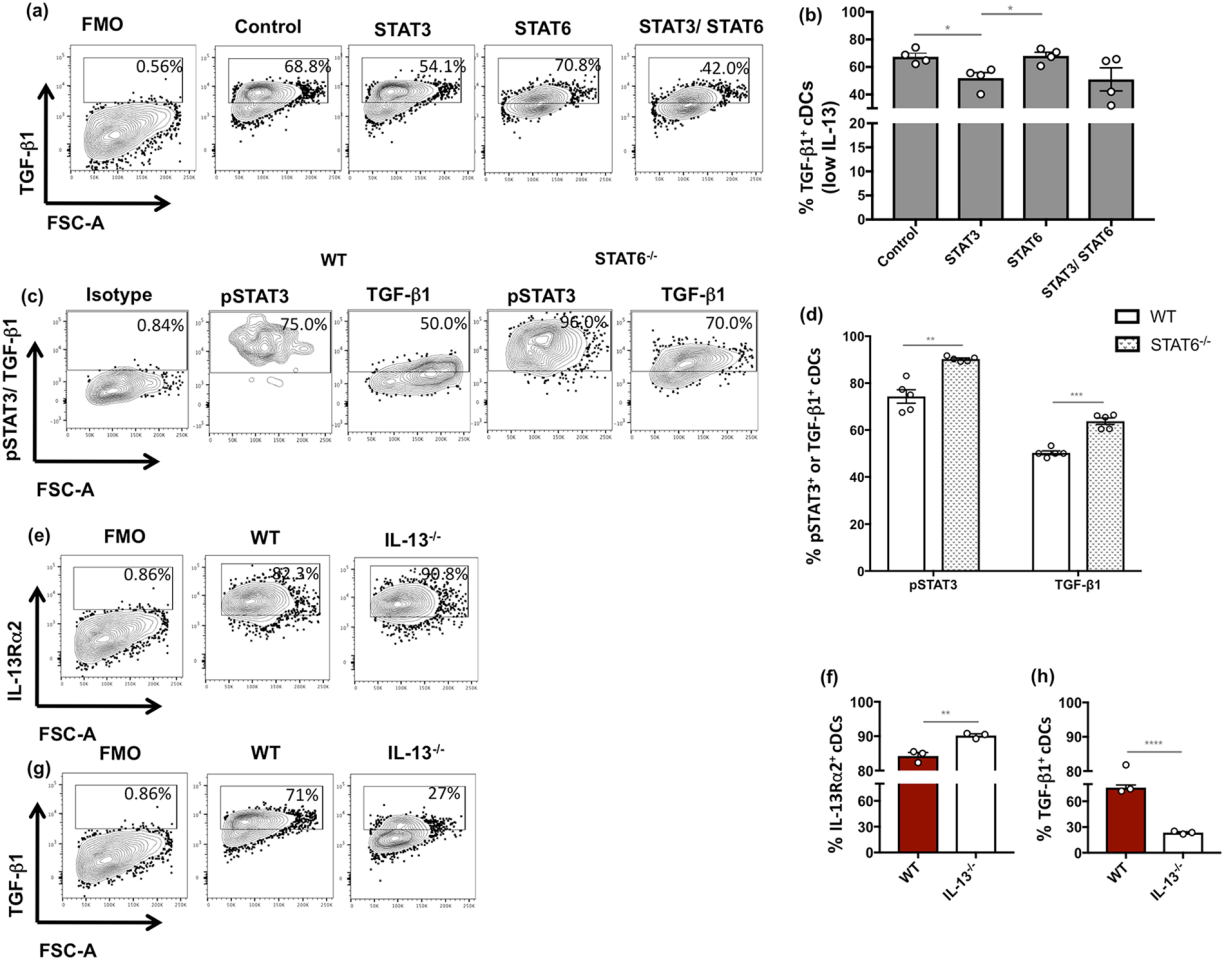
Evaluation of TGF-β1 on lung cDCs upon IL-13 stimulation *in vitro*, in the presence of STAT3 and STAT6 inhibitors or post rFPV vaccination. Unimmunised BALB/c lungs (n = 4) were treated with STAT3, STAT6 or combined STAT3/STAT6 inhibitors overnight, followed by 100 pg/ml of low IL-13 for 3 hours as described in methods. **(a**,**b)** Indicate representative FACS plots and graphs showing TGF-β1 expression in lung MHC-II^+^ CD11c^+^ CD11b^+^ CD103^−^ cDCs following *in vitro* STAT3 and STAT6 inhibition. **(c**,**d)** Indicate lung cDCs expressing TGF-β1 24 h post i.n. rFPV vaccination of STAT6^−/−^ and WT BALB/c mice (n = 5). **(e**,**f)** indicate IL-13Rα2 and **(g**,**h)** TGF-β1 expression on unimmunised IL-13^−/−^ and WT BALB/c mice (n = 3 per group). Error bars represent Standard Error of mean (SEM) and p values were calculated using paired Student’s t-test. *p < 0.05, **p < 0.01, ***p < 0.001, ****p < 0.0001. These experiments were repeated two to three times.

Since Fluidigm 48.48 Biomark analysis of rFPV vaccinated lung cDCs revealed that *Stat3* and *Tgfb1* gene expression were strongly correlated, next association of STAT3 activation/phosphorylation with TGF-β1 at the protein level was evaluated. *In vitro* inhibition studies under low IL-13 (100 pg/ml) stimulation revealed that STAT3 inhibition significantly down-regulated TGF-β1 expression in cDCs whilst STAT6 inhibition had no impact compared to the uninhibited control **(**Fig. 4a,b**)**. To understand the relationship between IL-13, IL-13Rα2, STAT3 and TGF-β1, when STAT6^−/−^ mice were vaccinated i.n. with rFPV (which induced low IL-13 at the vaccination site and enhanced IL-13Rα2 expression on lung cDCs, **(**Fig. 1)) and lung cDCs were assessed 24 h post vaccination, phosphorylated STAT3 (pSTAT3) and TGF-β1 were both up-regulated on STAT6^−/−^ cDCs compared to the wild type counterpart (*p* = 0.0038 and 0.0003 respectively) (Fig. 4c,d), suggestive of IL-13Rα2 signalling. Moreover, significant up-regulation of IL-13Rα2 (Fig. 4e,f) and down-regulation of TGF-β1 **(**Fig. 4g,h**)** were also observed in unimmunised IL-13^−/−^ cDCs compared to WT. Taken together these observations suggested that the measured TGF-β1 and IL-13Rα2 expression profiles were linked to IL-13 activity.

### IL-13Rα2 and IFN-γR were co-expressed on lung cDCs 24 h following i.n. rFPV vaccination

Our previous studies have shown that 24 h post viral vector vaccination, ILC1/ILC3- derived IFN-γ expression was inversely associated with ILC2-derived IL-13 at the vaccination site, which significantly impacted cDC recruitment^19,20^. Knowing that IFN-γ is a potent IL-13 inhibitor and can also mobilise IL-13Rα2 from intracellular compartments to the cell surface^29,32,33^, in this part of the study, the association of IFN-γR and IL-13Rα2 on lung cDCs, following i.n. rFPV vaccination was further investigated.

**Figure 5:**
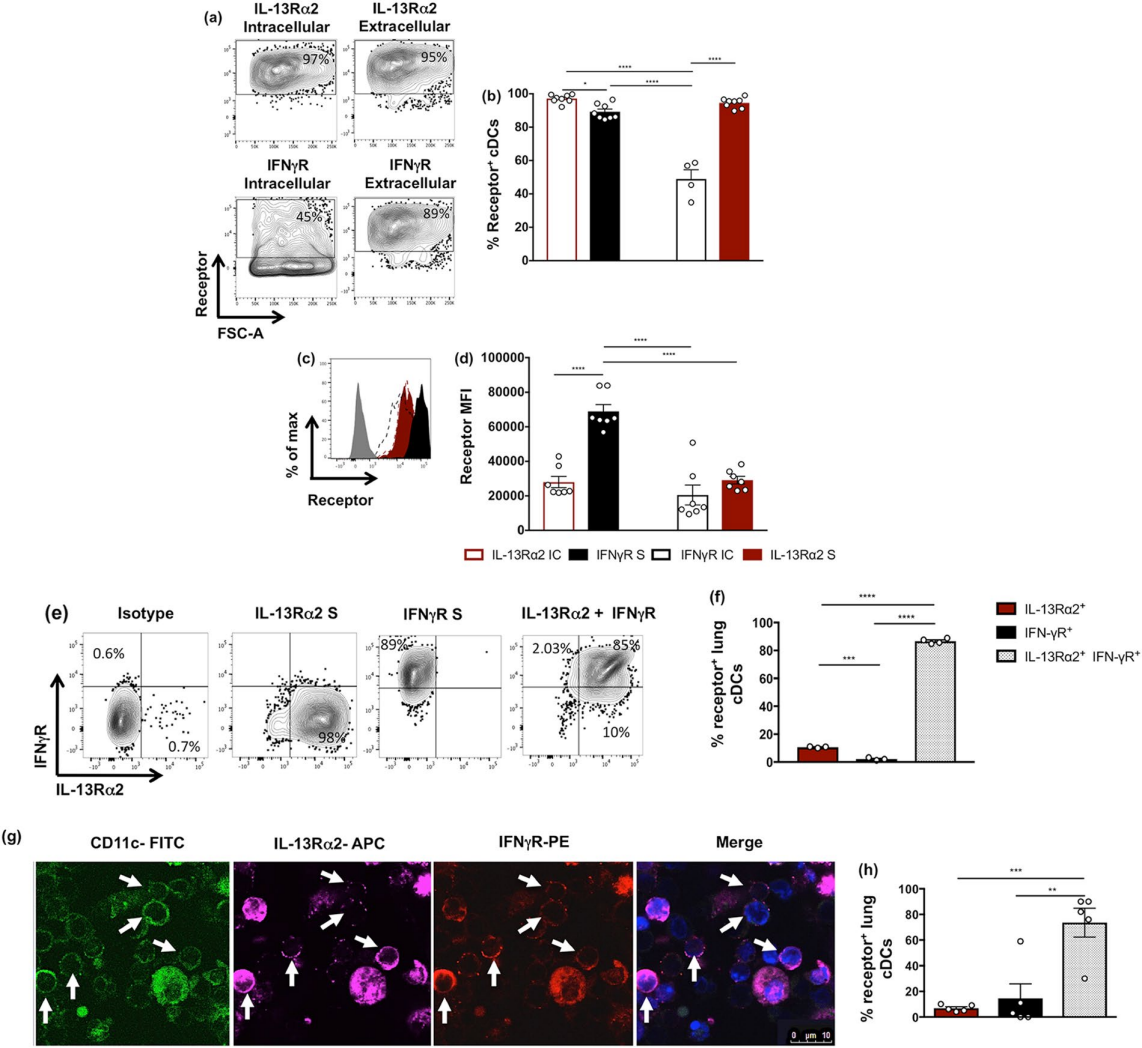
Evaluation of IL-13Rα2 and IFN-γR receptor expression on lung cDCs 24 h post rFPV vaccination. BALB/c mice (n = 5–8 per group) were intranasally (i.n.) immunised with rFPV. 24 h post delivery, lung cells were stained for MHC-II^+^ CD11c^+^ CD11b^+^ CD103^−^ cDCs to evaluate receptor expressions using flow cytometry as described in methods and Fig. S1a,b. **(a**,**b)** Representative flow cytometry plots and bar graph show the percentage of cDCs expressing intracellular and extracellular IL-13Rα2 and IFN-γR on lung cDCs. **(c**,**d)** Representative histogram plots and bar graph show a comparative extracellular IL-13Rα2 (solid red), intracellular IL-13Rα2 (dotted red), extracellular IFN-γR (solid black) and intracellular IFN-γR (dotted black) expression densities on lung cDCs from rFPV vaccinated mice compared to an isotype control (solid black). Error bars represent Standard Error of mean (SEM) and *p* values were calculated using One-way ANOVA followed by Tukey’s multiple comparison test and paired Student’s t-test. *p < 0.05, **p < 0.01, ***p < 0.001, ****p < 0.0001. **(e**,**f)** Representative flow cytometry plots and bar graph show single expression and co-expression of IL-13Rα2 and IFN-γR on lung cDCs. **(g**,**h)** Representative confocal microscopy images and bar graph show i.n. rFPV vaccinated (n = 5) lung cells expressing IL-13Rβ2 and IFN-γR at magnification x60 as described in methods and Fig. S7. Each white arrow indicates a single CD11c^+^ DC across all channels as well as merge image, co-expressing IL-13Rα2 and IFN-γR. These experiments were repeated three times.

Data revealed that following i.n. rFPV vaccination, differential IL-13Rα2 and IFN-γR expression levels were observed on lung cDCs **(**Fig. 5a–d). Specifically, the percentage of cDCs expressing intracellular IL-13Rα2 was significantly elevated compared to extracellular IFN-γR (*p* = 0.0228) (Fig. 5a,b). Alternatively, extracellular IL-13Rα2 was significantly elevated compared to intracellular IFN-γR (*p* < 0.0001), demonstrating an inverse correlation of the two receptors (Fig. 5a,b). When analysis was performed to evaluate whether lung cDCs co-expressed IL-13Rα2 together with IFN-γR following i.n. rFPV vaccination, flow cytometry data revealed that the majority of the cDCs were double positive for the two receptors (85%) **(**Fig. 5e,f). This was further substantiated by confocal imaging on lung CD11c^+^ DCs where ~75% of cells co-expressed IL-13Rα2 and IFN-γR (Figs. 5g,h and S7).

### rFPV, rMVA and Ad5 vaccinations differentially regulated IL-13 receptors, STAT3/STAT6 and IFN-γR on cDC 24 h post vaccination

**Figure 6:**
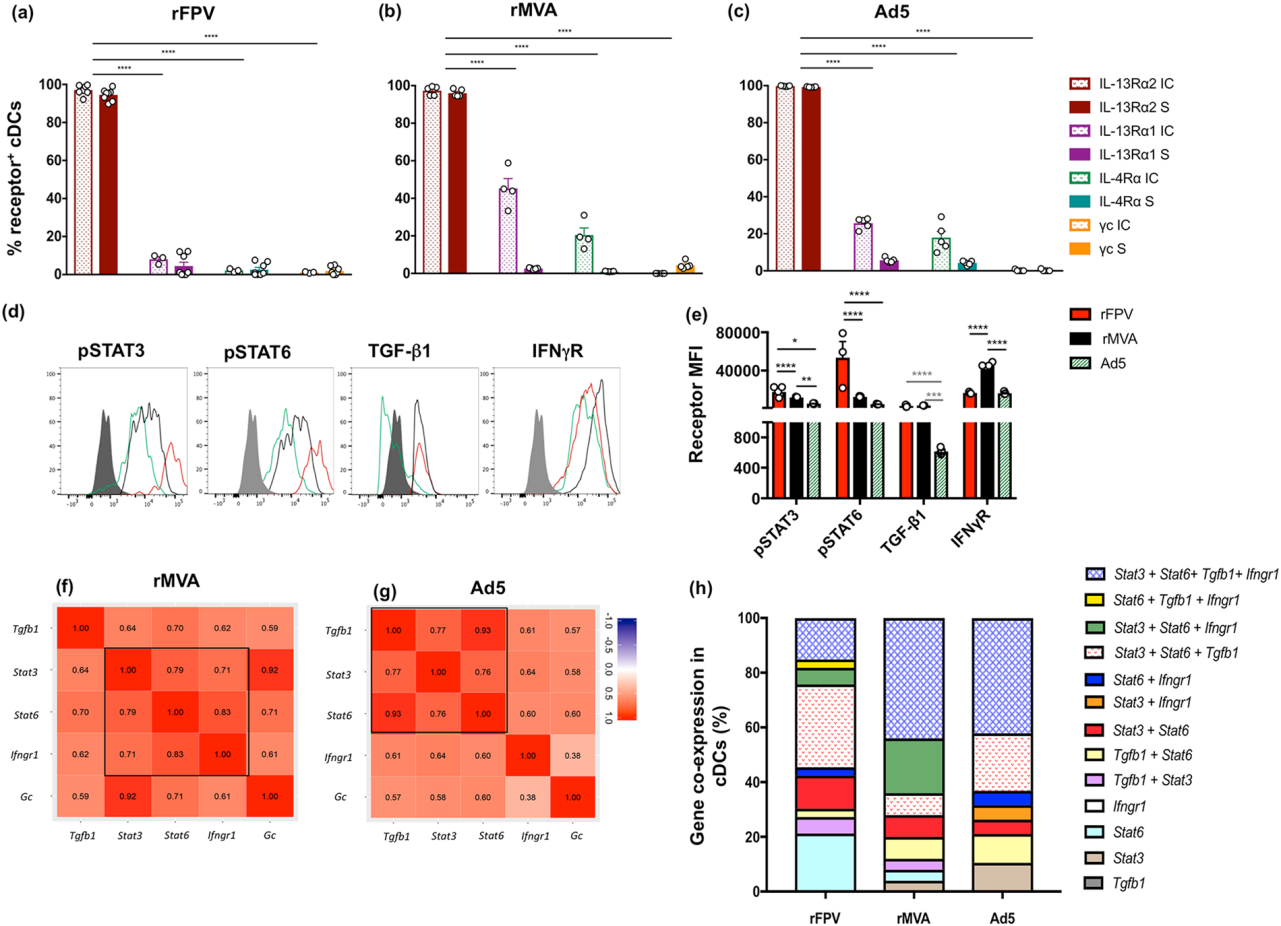
Evaluation of IL-4/IL-13 receptors and related genes on cDCs following different viral vector vaccinations. Graphs show evaluation of percentage of MHC-II^+^ CD11c^+^ CD11b^+^ CD103^−^ cDCs expressing IL-13Rα2, IL-13Rα1, IL-4Rα and γc receptors at the intracellular and extracellular levels 24 h post **(a)** rFPV, **(b)** rMVA and **(c)** Ad5 vaccination of BALB/c mice (n = 4–8), as described in methods and Fig. S8. **(d**,**e)** Representative histogram plots and bar graph show comparative extracellular pSTAT3, pSTAT6, TGF-β1 and IFN-γR expression densities on lung cDCs (n = 3–5) from rFPV vaccinated (red), rMVA (black), Ad5 vaccinated (green) compared to FMO controls for PE as a negative control (solid grey). These experiments were repeated three times. **(f**,**g)** Data show PCA (PC1 vs PC2) of *Tgfb1*, *Stat3*, *Stat6*, *Ifngr1* and *Gc* genes expressed on single lung cDCs following rMVA and Ad5 vaccination, post Fluidigm 48.48 Biomark analysis (Note that correlation analysis was performed between these genes, where values closest to 1.00 represent the strongest correlation). **(h)** Stacked bar graphs represent viral vector dependent *Stat3*, *Stat6*, *Tgfb1* and *Ifngr1* gene *co-*expression on cDCs following rFPV, rMVA or Ad5 vaccination using PCA and K-means clustering analysis as shown in Fig. S9. Each vaccine group represent 48 cells. Error bars represent Standard Error of mean (SEM) and p values were calculated using One-way ANOVA followed by Tukey’s multiple comparison test and paired Student’s t-test. *p < 0.05, **p < 0.01, ***p < 0.001, ****p < 0.0001. These experiments were repeated two times.

Knowing that different viral vectors can induce different ILC2-derived IL-13 levels and DC subsets at the vaccination site, which were associated with different vaccine- specific adaptive immune outcomes^20^, next the IL-4/IL-13 receptor expression and regulation on lung cDCs post i.n. rMVA and Ad5 delivery were compared to i.n. rFPV vaccination. Interestingly, even though all three vaccinations induced significantly elevated intracellular and extracellular expression of IL-13Rα2 on lung cDCs (95–98%), elevated IL13Rα1 and IL-4Rα expression (intracellular) were only detected in cDCs, following rMVA and Ad5 viral vector vaccination **(**Figs. 6a–c, S8). It was noteworthy that, both intracellular and extracellular expression of the latter two receptors was significantly lower (rFPV 1–12%, rMVA 1–58% and Ad5 2–30% respectively) compared to IL-13Rα2 (95–100%) **(**Figs. 6a–c, S8).

Interestingly, although lung cDCs obtained from rFPV, rMVA and Ad5 vaccine groups showed expression of *Stat6, Stat3, tgfb1 and Ifngr1* genes at a single cell level as well as at the protein level (pSTAT3, pSTAT6, TGF-β1 and IFN-γR) **(**Fig. 6d–g**)**, the expression profiles were significantly different between the three vaccine groups. Specifically, the expression of both pSTAT3 and pSTAT6 were found to be in the order of rFPV > rMVA > Ad5 **(**Fig. 6d**)**. The expression of TGF-β1 was similar in rFPV and rMVA, but significantly lower in Ad5 **(**Fig. 6d**)**. In contrast, in the context of IFN-γR expression, the order was found to be rMVA > rFPV > Ad5 **(**Fig. 6d**)**. At the mRNA level, rMVA and Ad5 cDCs showed a greater probability of *Stat3* and *Stat6* co-expression (79% and 76% respectively) compared to the rFPV group **(**Fig. 6f,g**)**. The probability of *Stat3* or *Stat6* co-expression together with *Ifngr1* was found to be in the order of rFPV (30%, 46%) < Ad5 (64%, 60%) < rMVA (71%, 83%) **(**Figs. 3b, 6f,g**)**. The probability of *Stat3* and *Tgfb1* co-expression was found to be very similar between rFPV (75%) and Ad5 (77%) cDCs **(**Figs. 3b, 6g**)**. However, *Stat6* and *tgfb1* co-expression profile was in the order of Ad5 > rMVA > rFPV (93%, 70%, 42% respectively) **(**Figs. 3b, 6f,g**)**.

To investigate differential regulation of *Stat3* and *Stat6* under different IL-13 conditions, a PCA was performed with respect to *Stat3, Stat6, Tgfb1* and *Ifngr1* **(**Figs. S9, 6h**)**. Distinct gene clusters with different combinations of the four genes were analysed as described in methods and Fig. S9. The proportion of each co-expression combination was represented as a stacked bar graph for each vaccine vector **(**Fig. 6h**)**, rFPV vaccination induced the highest proportion of cDCs expressing *Stat3* and *Stat6* together with *Tgfb1* (rFPV 30% vs rMVA 8%, Ad5 21%). Additionally, rFPV vaccinated cDCs expressing *Stat6* only (21%) and enhanced *Stat3* co-expression with other genes, indicated that the rFPV response was STAT3 dominant. Following rFPV vaccination, much lesser proportion of cDCs expressed *Stat3* and *Stat6* together with either *Ifngr1* (rFPV 6%, rMVA 20%, Ad5 0%) or *Tgfb1* and *Ifngr1* (rFPV 15%, Ad5 42%) **(**Fig. 6h**)**. In contrast, rMVA induced the highest proportion of cells expressing *Stat3* and *Stat6* together with either *Tgfb1* and *Ifngr1* (44%) or *Ifngr1* only (rMVA 20%, rFPV 6%, Ad50%). Compared to rFPV, rMVA induced lower proportion of cDCs expressing *Stat3/Stat6* in combination with *Tgfb1* (rMVA 8%, rFPV 30%). Following Ad5 vaccination, the majority of the cDCs expressed *Stat3* as well as *Stat6* along with *Tgfb1* and *Ifngr1* (Ad5 42%, rFPV 15%), comparable to the response exhibited with rMVA (44%). However, the proportion of Ad5 cDCs expressing *Stat3* as well as *Stat6* together with *Tgfb1* expression was intermediary to that of rFPV and rMVA, however much higher proportion of Ad5 cDCs co-expressed *Stat6* and *Tgfb1* (Ad5 10%, rMVA 8%, rFPV 3%). Also, Ad5 vaccinated cDCs exhibited a more predominant co-expression of other genes with *Stat6* compared to *Stat3*, indicating that unlike rFPV, the Ad5 response was STAT6 dominant **(**Fig. 6h**)**.

## Discussion

Asthma, allergy and vaccination studies have shown that lung cDCs are highly responsive to IL-13^8,20,34^. Interestingly, this study demonstrated that, IL-13Rα1 and IL-13Rα2 were differentially regulated on lung DCs in an IL-13 concentration and time dependent manner. At the steady-state (prior to immunization) significantly higher percentage of lung cDCs expressed IL-13Rα2 compared to IL-13Rα1. Furthermore, both these receptors were rapidly up-regulated on lung DCs upon IL-13 stimulation *in vitro* or 24 h post viral vector-based vaccination. Specifically, IL-13Rα2 expression was maintained under both low and high IL-13, whilst IL-13Rα1 was only observed under high IL-13 conditions, suggesting, in lung cDCs IL-13Rα2 was the primary sensor and mediator (master regulator) of IL-13 responses. Moreover, this was further substantiated by the presence of elevated stable IL-13Rα2 protein and minimal mRNA expression at 24 h post rFPV vaccination, elucidating a distinct inverse protein-mRNA regulation, unlike IL-13 mediated inflammatory conditions^2,35–38^. Non-linear protein-mRNA regulation of other proteins^39,40^, cytokines, including IL-13^41^ specifically, elevated protein and rapid mRNA degradation associated with protein stability have been previously documented^42–44^. Moreover, presence of minimal *Il13ra2* transcript levels in most mouse tissue types at steady-state^1,35,37,38,45^ (NCBI Gene ID: 16165) and in human cancers post-transcriptional regulation of IL-13Rα2 by alternative epigenetic pathways have also been reported^46^. Knowing that lung is continuously exposed to many environmental invasions (pathogens and allergens), the elevated stable IL-13Rα2 protein on lung DC may support the notion that, at the first line of defence (the lung mucosae), high affinity IL-13 receptor, IL-13Rα2 acts as the primary IL-13 sensor to mediate early IL-13 regulation/homeostasis and dysregulation of IL-13Rα2 could most likely be the cause of IL-13 mediated inflammatory disease.

Previous studies in our laboratory have shown that transient inhibition of IL-4/IL-13 signalling via STAT6 (using an rFPV based IL-4R antagonist adjuvanted HIV recombinant viral i.n. rFPV prime/i.m. rMVA or rVV boost vaccination strategy) or transient sequestration of IL-13 at the vaccination site (using IL-13Rα2 adjuvanted HIV recombinant viral i.n. rFPV prime/i.m. rMVA or rVV boost vaccination strategy) can induce high avidity/poly-functional mucosal and systemic T cells with better protective efficacy^15,17^, which was associated with elevated cDC recruitment^17,19^. These studies also showed that IL-13 was necessary for effective antibody differentiation^15^, which was regulated via a STAT6 independent pathway^28^. When trying to unravel how IL-13 modulated these different vaccine-specific outcomes current study revealed that, i) under low IL-13 conditions/rFPV vaccination (which induced low IL-13 at the lung mucosa), IL-13Rα2 expression was up-regulated on DC; ii) under low IL-13/STAT3 inhibition IL-13Rα2 expression was up-regulated whilst TGF-β1 was down-regulated on lung DCs, as opposed to STAT6 inhibition; iii) Moreover, up-regulation of phosphorylated STAT3 and TGF-β1 was detected on STAT6^−/−^ cDCs post rFPV vaccination. There findings collectively suggested that, under low IL-13 environments, cDCs most likely mediated IL-13 activity exclusively via IL-13Rα2 by promoting STAT3/TGF-β1 activation, which was consistent with other findings^2,27^. Also, the intriguing enhanced phosphorylated STAT6 expression on lung cDCs under low IL-13 signified a co-regulation of STAT3/STAT6 during this process. However, performing vaccination studies in IL-13Rα2^−/−^ mice, to establish the ‘direct’ association of IL-13Rα2 signalling via STAT3 to induce TGF-β1 would have added great value to our findings and this warrants further investigation.

**Figure 7:**
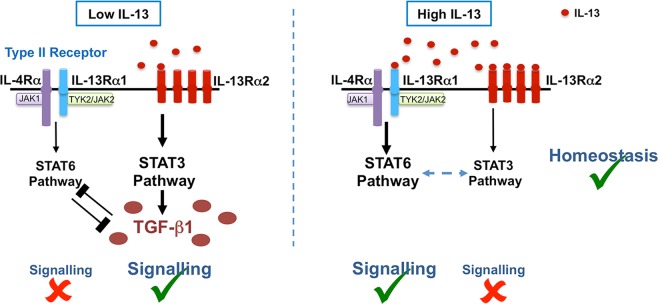
Proposed dual action of IL-13Rα2/STAT3 associated with IL-13 regulation in lung cDCs following viral vector-based vaccination. Under low IL-13 conditions, IL-13Rα2 mediates IL-13 signalling via STAT3 to promote TGF-β1 expression. In contrast, under high IL-13 conditions, IL-13Rα1 mediates IL-13 signalling via STAT6, and IL-13Rα2 acts to sequester excess IL-13 in the milieu (does not signal) and activates STAT3, to maintain IL-13 homeostasis at the vaccination site. Findings in the literature and our current study indicate that STAT3 and STAT6 can co-regulate each other to prevent immune dysregulation under both these conditions.

Under high IL-13, in addition to our study reconfirming the well-characterised IL-13Rα1/IL-4Rα signalling via STAT6^1^, we also showed regulation of IL-13Rα2 and co-expression of both IL-13Rα1 and IL-13Rα2 on lung DCs. These observations suggested that i) unlike low IL-13 conditions, DCs responded to high IL-13 predominantly via IL-13Rα1/STAT6 pathway and ii) under high IL-13 conditions, IL-13Rα2 likely regulated IL-13 in a STAT3 dependent manner. Moreover, the unexpected up-regulation of IL-13Rα2 under high IL-13 and dual STAT3/STAT6 inhibition also suggested the possible involvement of STAT3-independent IL-13Rα2 signalling mechanisms, similar to IL-4 signalling via STAT1 and STAT5^47^ (redundancies built into the system to regulate IL-13). In inflammatory diseases and high IL-13 conditions, IL-13Rα2 is recognized to be a decoy receptor that sequesters excess IL-13^35,37^. Interestingly, rMVA and Ad5 vaccination, which promoted high IL-13^20^, expressed *Stat6* mRNA and phosphorylated STAT6 on lung cDCs, associated with IL-13Rα1 signalling together with *Stat3* and phosphorylated STAT3 activation. Knowing that IL-13Rα2 can regulate IL-4Rα/STAT6^48^, promote TGF-β1 expression and latter can also regulate STAT6^49^, we propose that elevated IL-13 in the milieu post viral vector vaccination (i) can activate IL-13Rα1/STAT6 signalling whilst promoting IL-13 sequestration by IL-13Rα2 in a STAT3 dependent manner on lung cDCs and (ii) IL-13Rα2 can also regulate STAT6 in a STAT3 dependent manner, to prevent excessive IL-13 signalling on lung cDCs to maintain homeostasis at the lung mucosae **(**Fig. 7**)**.

Studies have shown that STAT6 and STAT3 can be differentially regulated, according to the state of viral infection/vaccination. Specifically, in the context of viral vector-based vaccination whilst IL-13/STAT6 signalling has been shown to dampen effective antiviral immunity^13,28^, however in acute and primary viral infections, it has shown to improve antiviral immunity^50,51^. This study showed that viral vector induced IL-13 “level” in the cell milieu significantly altered the STAT3/STAT6 equilibrium. Specifically, rFPV vaccination, associated with low ILC2-derived IL-13 at the vaccination site^18,52^, exhibited enhanced STAT3 expression (both at mRNA protein levels), which correlated with TGF-β1 on lung cDCs, suggesting a positive regulation of IL-13Rα2/STAT3 by TGF-β1. In contrast, a negative association of *Stat3* with *Ifngr1*, was confirmed by the inverse correlation and co-expression pattern of IFN-γR with IL-13Rα2 on cDCs, suggesting that IL-13Rα2 could be negatively regulated by IFN-γ, under low IL-13 conditions, which is in agreement with studies by Daines *et al*.^29^.

Data revealed that as opposed to rFPV vaccinated lung cDCs, rMVA vaccinated lung cDC (associated with high ILC2-derived IL-13 at the vaccination site^20^), exhibited both STAT3 and STAT6 expression, associated with an IFN-γR expression bias (both at the mRNA and protein levels). Interestingly, Ad5 vaccinated lung cDC, (associated with moderate ILC2-derived IL-13, intermediate of rFPV and rMVA^20^), showed higher association of STAT3 with IFN-γR compared to TGF-β1 (both the mRNA and protein levels). Knowing that IFN-γ can regulate IL-13 responses^30^, these observations indicated that following viral vector-based vaccination, at the cDC level the differential environmental immune responses to IL-13 are not only determined/regulated by STAT3/STAT6, but also by TGF-β1 and IFN-γ either collaboratively or independently, which was consistent with cancer/inflammation studies^53–56^. Interestingly, rapid STAT3 activation has shown to control some viral infections^51,57,58^ whilst, STAT6 independent mechanisms have also been associated with effective antibody differentiation^28^. Moreover, IL-13 mediated enhanced IFN-γ signalling has been shown to exacerbate respiratory viral infections^31,59^. Collectively, our findings propose the notion that in the context of viral vector-based vaccination and recruitment of DCs, vectors that promote low ILC2-derived IL-13, induce IL-13Rα2 signalling and STAT3/TGF-β1 activation, are associated with effective T and B cell immune outcomes. In contrast, vectors that promote high ILC2-derived IL-13 induce IL-13Rα1/STAT6 signalling and elevated IFN-γ activity, lead to suboptimal vaccine-specific T cell outcomes. This may explain why in a prime-boost vaccine modality, choice of viral vector or adjuvant used in a ‘prime’ can crucially impact the vaccine-specific functional CD8 T cell avidity^14^, (knowing that booster vaccination mainly expands the initial high or low avidity T cell subset generated during priming)^17^.

In conclusion, our current study demonstrated a dual role of IL-13Rα2/STAT3 in IL-13 regulation at the lung mucosae. Specifically, under viral vaccination-induced low IL-13, IL-13Rα2 functioned as a signalling receptor on lung DCs, whilst, under high IL-13, mediated homeostasis by sequestration of excess IL-13 in the cell milieu, both involving STAT3 activation and co-regulation of STAT3 and STAT6 (Fig. 7). Hence, fully understanding these IL-13, STAT3/STAT6 regulatory paradigms, have high potential to help design more efficacious vaccines against chronic pathogens and also therapies against other IL-13 related diseases.

## Materials and Methods

### Mice

Pathogen-free 6–8 weeks old female wild type BALB/c, IL-13^−/−^ and STAT6^−/−^ mice on a BALB/c background were purchased from the Australian Phenomics Facility, The Australian National University (ANU). All animals were maintained, monitored daily, euthanized by cervical dislocation and experiments were performed in accordance with the Australian NHMRC guidelines within the Australian Code of Practice for the Care and Use of Animals for Scientific Purposes and in accordance with guidelines approved by the ANU Animal Experimentation and Ethics Committee (AEEC), protocol number A2014/14 and A2017/15.

### Immunisation and preparation of lungs

BALB/c mice were intranasally immunised with 1 × 10^7^ plaque forming units (pfu) of rFPV, rMVA, or 2 × 10^7^ pfu of Ad5. Mice were vaccinated with a volume of 10 µl per nostril (total 20 µl) under mild isofluorane anaesthetic. rFPV and rMVA were sonicated thrice for 15 seconds in ice at 50% capacity using Branson Sonifier 450 immediately prior to vaccination.

### Evaluation of lung DCs and IL-4/IL-13 and IFN-γ receptors using Flow cytometry

Lung tissues were collected 24 h post vaccination as described in Li *et al*.^18^. 2 × 10^6^ cells from each sample were blocked with anti-mouse CD16/CD32 antibody (BD Biosciences, USA) for 20 min at 4 °C and cells were surface stained with APC-conjugated anti-mouse MHCII I-Ad (e-Biosciences, USA), biotin-conjugated anti- mouse CD11c (N418 clone, Biolegend, USA), followed by streptavidin Brilliant violet 421 (Biolegend, USA), anti-mouse CD11b AlexaFluor 700 (M1170 clone, Biolegend, USA) and anti-mouse CD103 FITC (2E7 clone, e-Biosciences, USA) for 30 min on ice as previously described in^20^. Cells were additionally extracellularly or intracellularly stained with anti-mouse IL-4Rα (CD124) PE (I015F8 clone, Biolegend, USA), anti-mouse IL-13Rα1 (CD213a) PE (13MOKA clone, e-Biosciences, USA), Biotin-conjugated anti-mouse IL-13Rα2 (110815 clone, R&D systems, USA), followed by streptavidin PE (Biolegend, USA), anti-mouse γc (CD132) PE (TUGm2 clone, Biolegend, USA) and biotin-conjugated anti-mouse IFN-γRa chain (CDw119) (2E2 clone, Biolegend, USA), followed by streptavidin PE (Biolegend, USA). For intracellular staining, cells were fixed using Fixation buffer (Biolegend, USA) for 10 minutes at 4 °C followed by permeabilisation using 1x Intracellular staining permeabilisation wash buffer (Biolegend, USA) for 10 minutes at 4 °C prior to intracellular staining. Cells were fixed using 1.5% paraformaldehyde followed by resuspension in PBS and analysed using BD LSRII flow cytometer Becton Dickinson, San Diego, CA). 5 × 10^5^ events per sample were acquired and results were analyzed using FlowJo software v10.0.7.

### *In vitro* STAT3 and STAT6 inhibition assays

Unimmunised BALB/c lung suspensions were treated with either 100 nM of small molecule STAT6 inhibitor (Axon Medchem) or 20 μM Stattic (small molecule STAT3 inhibitor) in PBS overnight followed by low (100 pg/ml) or high (10,000 pg/ml) IL-13 stimulation for 3 h or 0.5 h before evaluation of IL-4 and IL-13 receptor expression on lung DCs using flow cytometry as described above. Biologically relevant inhibitor concentrations were used in this study as reported previously^28,60^.

### Immunofluorescence assays

Single cell suspensions of lungs were washed to remove media and blocked with anti-mouse CD16/CD32 Fc Block antibody (BD Biosciences, USA) for 20 min at 4 °C and cells were surface stained with FITC- conjugated anti-mouse CD11c (N418 clone, Merck, Germany), anti-mouse IL-4Rα (CD124) PE (I015F8 clone, Biolegend), anti-mouse IL-13Rα1 (CD213a) PE (13MOKA clone, e-Biosciences, USA), Biotin-conjugated anti-mouse IL-13Rα2 (110815 clone, R&D systems, USA), followed by streptavidin APC (Biolegend, USA) and biotin-conjugated anti-mouse IFN-γRα chain (CDw119) (2E2 clone, Biolegend, USA), followed by streptavidin PE (Biolegend, USA). Cells were fixed using 1.5% Paraformaldehyde (Biolegend, USA) and suspension cells were centrifuged onto Poly-L-Lysin (Sigma, USA) coated glass cover slips. Cover slip containing cell pellet was covered with 10 μl of Antifade Vectashield mounting medium with or without 4′,6-diamidino-2-phenylindole (DAPI) from Vector Laboratories, USA and mounted onto a clean glass slide. Slides were imaged and analysed using Leica TCS SP5 confocal microscope (Leica, Germany) at 60x magnification. DAPI^l^°^w^ CD11c^+^ cells were identified as viable lung DCs for receptor expression. To quantify receptor co-expression, each CD11c^+^ DC double positive for a given receptor combination (IL-13Rα1 and IL-13Rα2, IL-4Rα and IL-13Rα2, or IL-13Rα2 and IFN-γR) was identified and quantified per imaged area as described in Fig. S7. Proportion of each receptor combination was calculated as a percentage of the total number of viable DCs per imaged area. Data were represented as an average of 5 imaged areas from each experiment. To quantify IL-13/IL-4 receptor intensity, ImageJ software v 1.52e (for Windows) was used. During this process, DAPI^l^°^w^ CD11c^+^ cells expressing the receptor of interest were identified **(**Fig. S3**)**. Next, each cell was identified as a region of interest (ROI) and the software generated integrated density of the ROI was used to calculate receptor intensity as; IL-13/IL-4 receptor intensity = (Integreated density of ROI/Area of ROI).

### cDC sorting for Fluidigm 48.48 Biomark and qPCR assays

Single (n = 48 per vaccine group) or 500 cDCs were sorted into 5 µl or 25 µl pre-amplification mixture respectively using a BD FACS Aria II cell sorter, using the gating strategy described in **(**Fig. S4**)**. The pre-amplification mixture contained 2x reaction buffer, SuperScript® III RT/Platinum® Taq Mix, 0.2x pooled assays, SUPERase• In™ RNase Inhibitor and DEPC treated water per well.

Sorted cDCs in pre-amplification mixture were centrifuged at 1454 × g to release mRNA as previously described^61^. The cDNA was synthesised using thermo-cycling program: 1x cycle of 50 °C for 15 minutes, 95 °C for 2 minutes followed by 14–20 cycles (for single or 500 cells) of 95 °C for 15 seconds and 60 °C for 4 minutes, followed by storing samples at −20 °C until use.

### Real-time quantitative PCR (RT-qPCR) analysis of IL-4/IL-13 receptors

RT-qPCR for 500 cells was performed using TaqMan qPCR mix (containing 1 μL of each gene expression assay (IL-4/IL-13 primers - Table S1), 5 μL of 2X TaqMan Universal PCR master mix, 1 μL cDNA and 4.5 μL of DEPC treated water), using a 7900HT thermocycler program: 50 °C for 2 minutes, 95 °C for 10 minutes, and 45 cycles of 95 ^o^C for 15 seconds and 60 °C for 1 minute. The targeted primer-probe FAM fluorescence was detected by normalising to ROX (6- carboxy-X-rhodamine) intensity. SDS 2.4 for Windows software was used to obtain the cycle threshold (Ct) values (ranging from 0 to 45) and the mRNA amplification profiles. Ct values were subject to quality control using SDS 2.4 analysis software where, 0 indicated a high expression and values closer to 45 indicated low expression levels.

### Fluidigm 48.48 Biomark gene expression assay

Fluidigm 48.48 gene expression assay was performed as previously described^61^. Briefly, prior to loading the integrated fluidic chip (IFC) (Fluidigm), the cDNA was diluted 1:1 cDNA:DEPC treated water. Following chip priming, 2.5 μL of diluted cDNA (in DEPC water) and 0.25 μL of 20X GE Sample Loading Reagent was loaded onto the sample side of the chip. Subsequently, 2.5 μL of each gene expression assay **(**Fig. S9**)** and 2.5 μL of 20X GE Assay Loading Reagent was loaded onto the assay side of the IFC. Next, the IFC was loaded onto the IFC Controller MX and gene expression assay was performed and analysed using the GE 48.48 Standard.pcl program on the Fluidigm Biomark^TM^. The fluorescence values obtained from the Fluidigm Biomark^TM^ were normalised to ROX (6- carboxy-X-rhodamine) intensity. Ct values (ranging from 0 to 40) were subject to quality control using the Biomark Real-time qPCR analysis software where, 0 indicated a high expression and values closer to 40 indicated low expression levels. Binary analysis was performed to determine the proportion of cells expressing a certain gene using RStudio and Microsoft Excel 2016 software and analysed using GraphPad Prism 7.0.

### Statistical analysis

Lung MHC-II^+^ CD11c^+^ CD11b^+^ CD103^−^ cDC proportions were represented as a percentage of total MHC-II^+^ CD11c^+^ DCs and receptor proportions were calculated as a percentage of parent cDC population as described in^20^. The *p*-values were calculated using either two-tailed, paired parametric Student’s t-test or Ordinary One-way ANOVA with Tukey’s multiple comparison post-test. Gene expression was first analysed as percentage of cDCs expressing a gene of interest. For each gene of interest, the Ct value for the housekeeping gene (*l32*) was subtracted from each sample Ct value to determine ΔCt, and the gene expression level was calculated as 40^−ΔCt^ or 45^−ΔCt^. All experiments were repeated minimum two times. Principal Component Analysis (PCA) was performed to analyse the relationship between the genes, using a correlation matrix created using Spearman’s rho (ρ) as described previously^61^. To determine the co-expression profile with respect to only *Stat3, Stat6, tgfb1* and *Ifngr1*, following PCA, a k-means clustering algorithm using RStudio was used to identify clusters. To determine statistical significance with respect to co-expression studies, a Fisher’s exact test was implemented with False Discovery Rate (FDR) correction.

## Supplementary information

Supplementary Figures.

## Data Availability

The authors declare that all data supporting this study are available in the paper and Supplementary files.
