# Supplementary material for: Unique IL-13Rα2/STAT3 mediated IL-13 regulation detected in lung conventional dendritic cells, 24 h post viral vector vaccination
Source: Sci Rep. 2020 Jan 23;10:1017. doi: 10.1038/s41598-020-57815-z (PMC6978450; doi:10.1038/s41598-020-57815-z)
Supplement: Supplementary file 1 — Supplementary Figures. [file 41598_2020_57815_MOESM1_ESM.pdf]

Unique IL-13Rα2/STAT3 mediated IL-13 regulation detected in lung conventional dendritic cells, 24 h post viral vector vaccination.

Sreeja Roy[1,¶], Ho-Ying Liu[1,¶], Muhammad Irwan Jaeson[1], Lachlan Paul Deimel[1], Charani Ranasinghe[1]*

Running title: **Viral vector induced differential IL-13 signalling via IL-13Rα2, STAT3 and TGF-β1**

*Correspondence: charani.ranasinghe@anu.edu.au

Telephone: +61 2 6125 4706

[1]Molecular Mucosal Vaccine Immunology Group, Department of Immunology and Infectious Disease, The John Curtin School of Medical Research, The Australian National University, Canberra ACT 2601, Australia.

[¶]Authors contributed equally to the work.

Supplementary Fig S1(a and b)

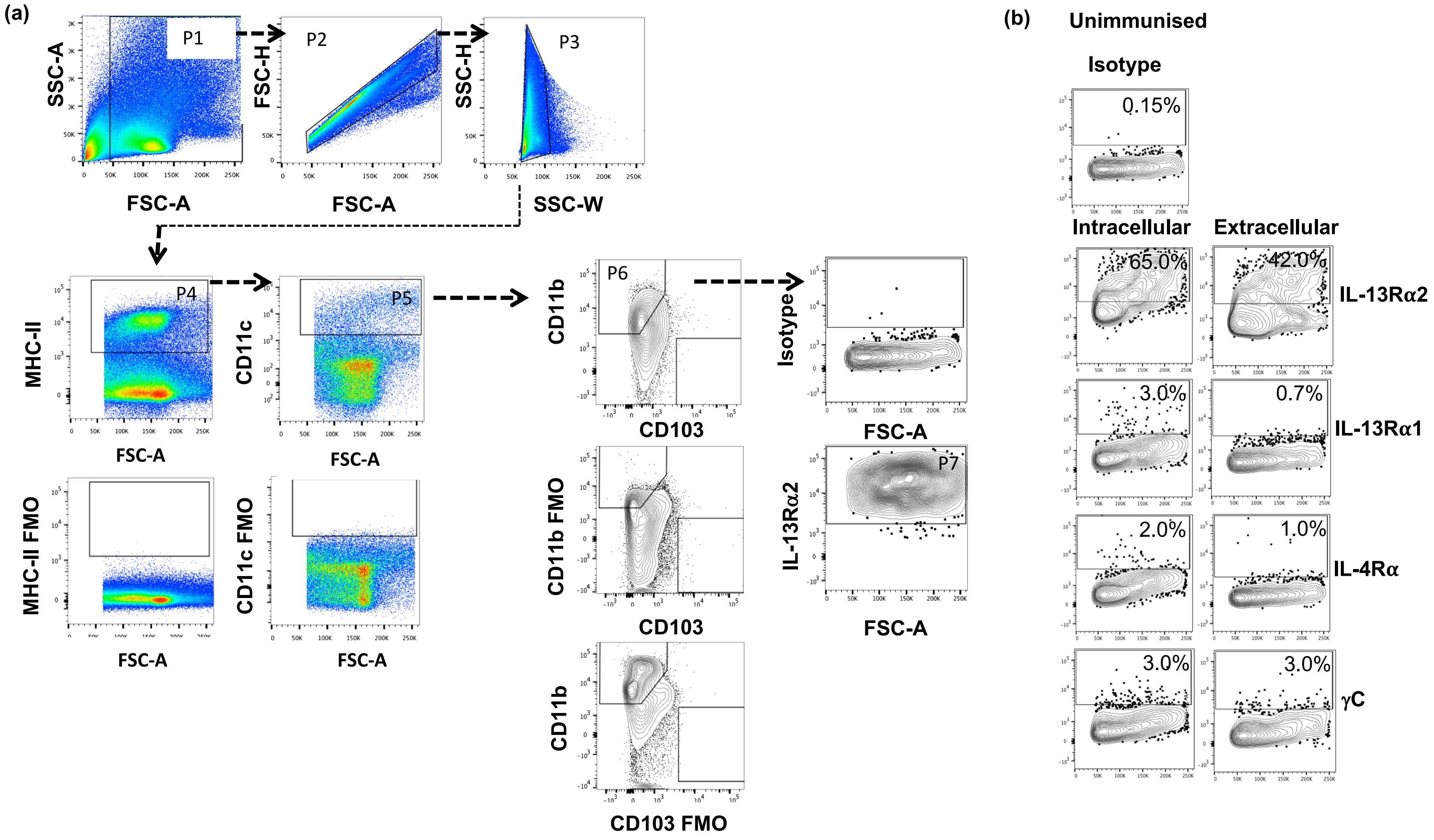

Fig S1(a and b). Flow cytometry gating for lung cDCs and IL-4/IL-13 receptors 24 h following i.n. rFPV immunisation. **(a)** Plots show viable cells (P1) after gating on single cells based on forward scatter (FSC-H and FSC-A; P2) and side scatter (SSC-H and SSC-W; P3) were then gated on MHC-II-I-Ad^{+} (P4) and analysed for CD11c expression compared to FMO controls. Total DCs (MHC-II-I-A^{d+} CD11c^{+} - P5) were further gated on CD11b^{+} CD103^{-} (P6) using appropriate FMO controls for each marker. Receptor positive cells (P7) were gated based on isotype controls specific for the viral vector. (Note that to avoid breaking the CD11b population in half, the FMO was set to include CD11b^{low}, CD11b^{int} and CD11b^{hi} populations, even though IL-13Rα2 expression was mainly associated with CD11b^{int} and CD11b^{hi} populations). **(b)** Representative flow cytometry plots show intracellular and extracellular IL-13Rα2, IL-13Rα1, IL-4Rα and γc expressions on lung cDCs from unimmunised BALB/c mice.

Supplementary Fig S1c

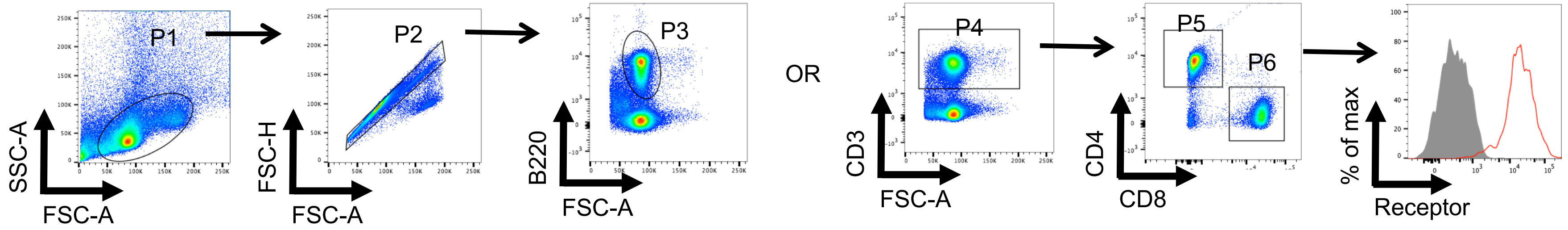

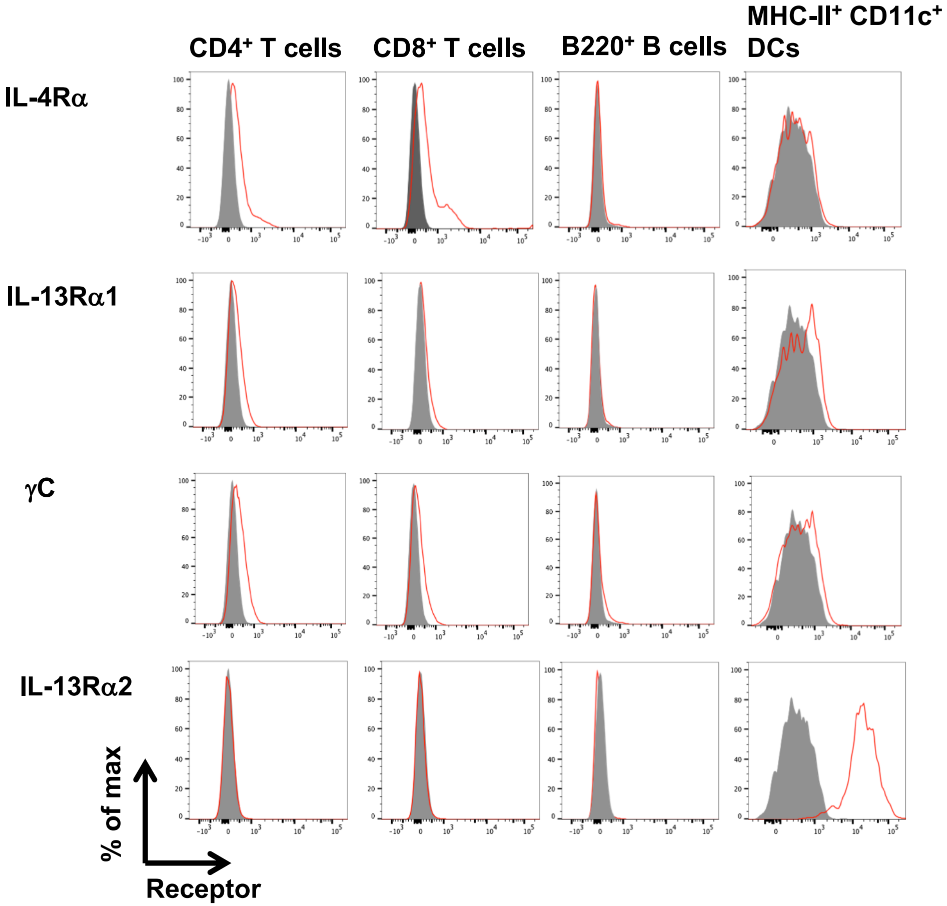

Fig S1c. Evaluation of IL-4 and IL-13 receptor expression on lung lymphocytes and DCs 24h following rFPV vaccination. BALB/c mice n=5 were i.n. vaccinated with rFPV and 24h post lungs were prepared as described in methods. (Top Panel) $B220^{+}$ B cells (P3) were gated from Single lymphocytes (P2) and $CD4^{+}$ T cells (P5) and $CD8^{+}$ T cells (P6) were gated from $CD3^{+}$ T cells (P3), following gating on single lymphocytes (P2). MHC-II^{+} $CD11c^{+}$ DCs were gated following strategy shown in Fig S1A and B. (Bottom panel) Flow cytometry analysis was performed and representative histogram plots show geometric mean intensities for IL-4Rα, IL-13Rα1, γc and IL-13Rα2 (red line) against the isotype control (solid grey) on lung $CD4^{+}$ T cells, $CD8^{+}$ T cells, $B220^{+}$ B cells and MHC-II^{+} $CD11c^{+}$ DCs.

Supplementary Fig S1d

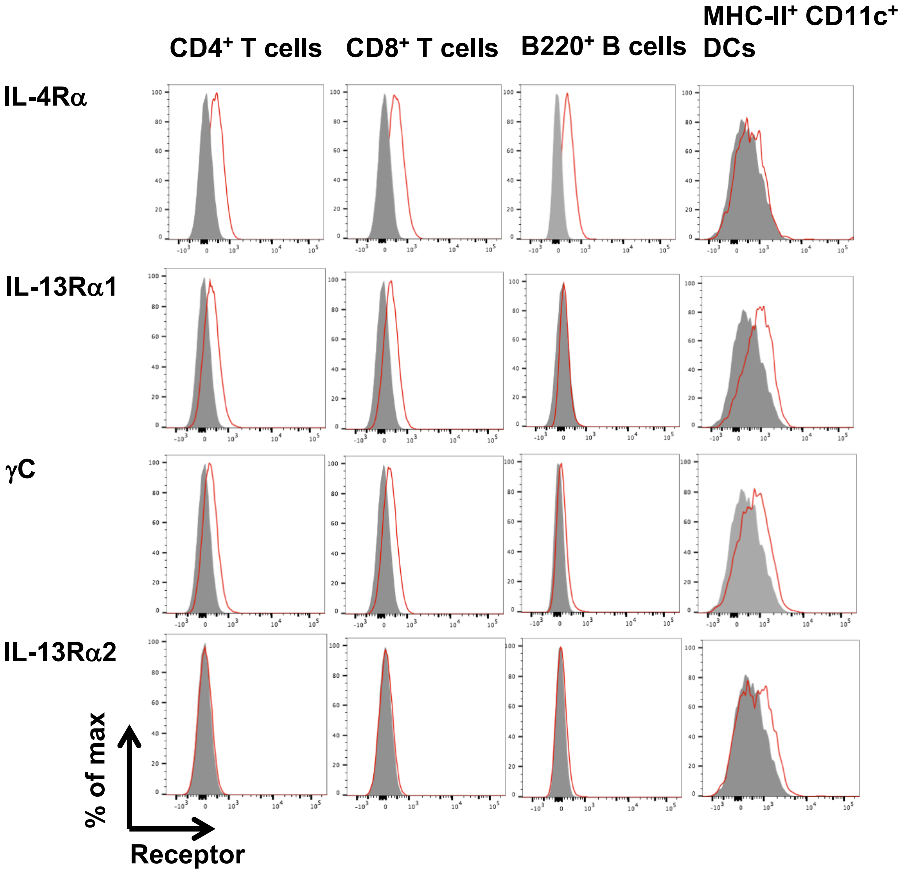

Fig S1d. Evaluation of IL-4 and IL-13 receptor expression on splenic lymphocytes and DCs. Unimmunised BALB/c mice n=5 were used to obtain spleens and single cell suspensions were prepared to stain for lymphocytes and DCs as described in methods. CD4^{+} T cells, CD8^{+} T cells and B220^{+} B cells were gated as shown in Fig S1C and MHC-II^{+} CD11c^{+} DCs were gated following strategy shown in Fig S1A and B. Flow cytometry analysis was performed and representative histogram plots show geometric mean intensities for IL-4Rα, IL-13Rα1, γc and IL-13Rα2 (red line) against the isotype control (solid grey) on splenic CD4^{+} T cells, CD8^{+} T cells, B220^{+} B cells and MHC-II^{+} CD11c^{+} DCs.

Supplementary Fig S1e

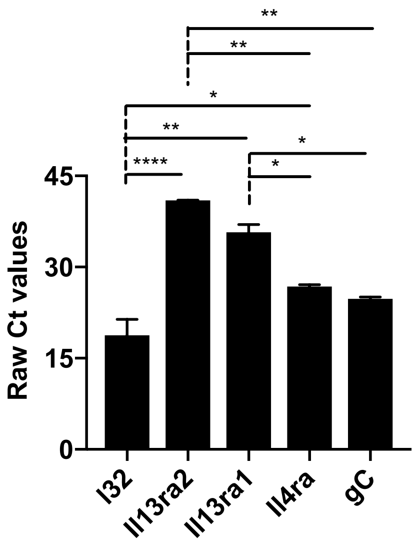

Fig S1e. Evaluation of IL-4/ IL-13 receptor mRNA expression on lung cDCs at 24 h post rFPV vaccination. BALB/c mice lungs (n=3) were harvested at 24 h post rFPV vaccination and single cell suspensions were FACS sorted for 500 MHC-II^{+} CD11c^{+} CD11b^{+} CD103^{-} cDCs to evaluate IL-4 and IL-13 receptors at the mRNA level using qPCR as described in methods. Bar graphs represent raw Ct values for all receptors and house-keeping gene, Ribosomal protein L32 (*l32).* Note that high Ct indicates low mRNA expression. Error bars represent Standard Error of mean (SEM) and *p* values were calculated using One-way ANOVA followed by Tukey's multiple comparison test. *$p<0.05$, **$p<0.01$, ***$p<0.001$, ****$p<0.0001$. Experiments was repeated two times.

Supplementary Fig S2

(a) DAPI only background control (for immunoflourescence on rFPV vaccinated murine lung cells)

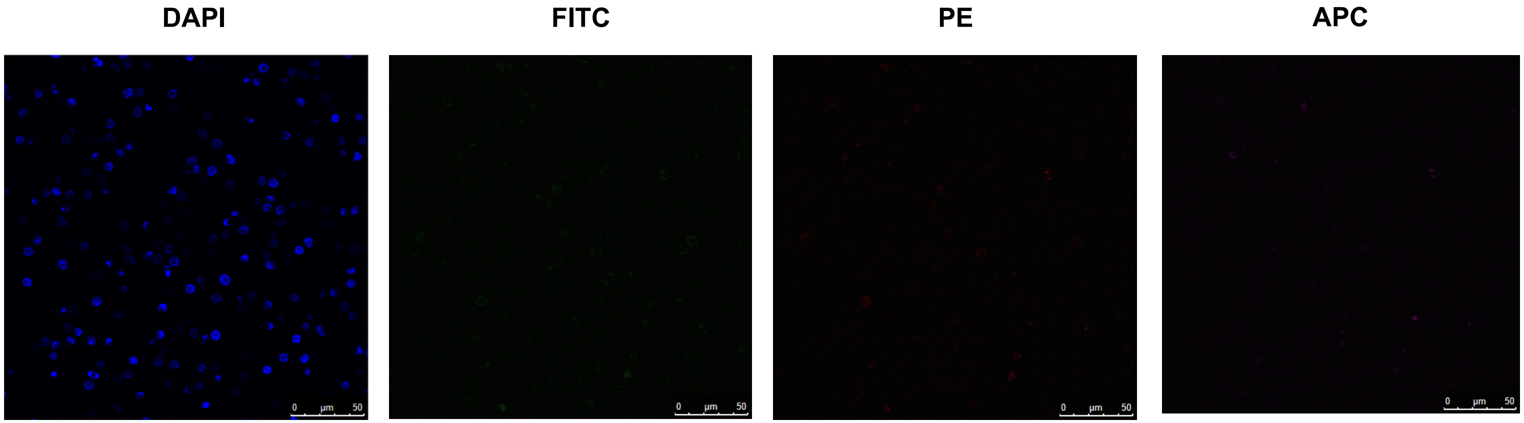

(b) DAPI only background control and unstained control (for in vitro IL-13 stimulation studies)

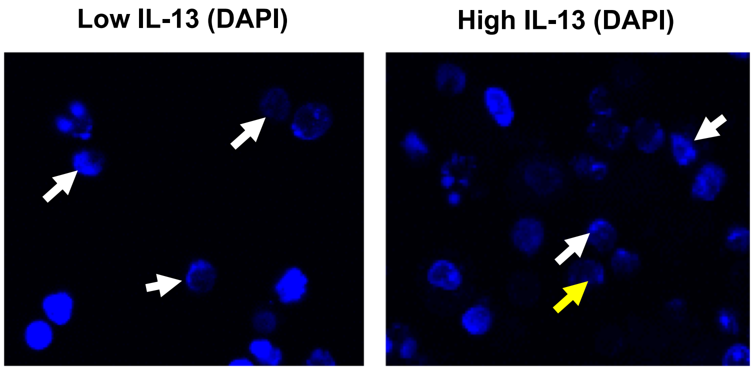

(c)

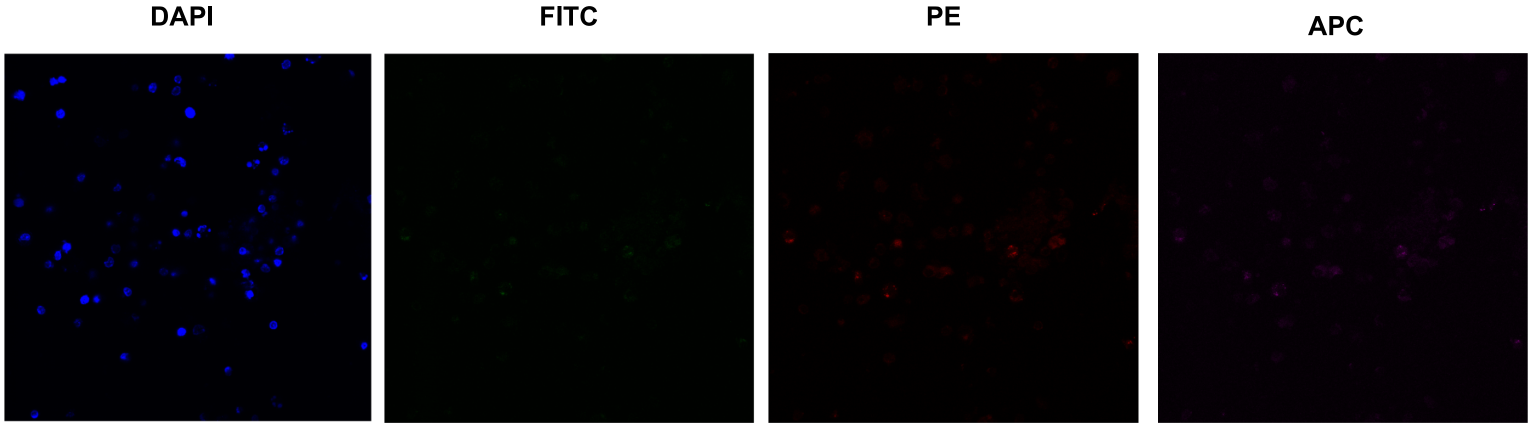

Fig S2. Immunofluorescence imaging of lung CD11c^{+} DC negative controls for receptors using confocal microscopy. Representative confocal microscopy images of lung cells stained with DAPI to identify viable cells unstained for receptor antibodies, showing negative controls for the FITC, PE and APC channels from **(a)** rFPV vaccinated BALB/c (n=5), **(b and c)** unimmunised BALB/c (n=5) with no stimulation, low (100pM) and high (10000pM) IL-13 . White arrows indicate CD11c^{+} DCs co-expressing IL-13Rα2 and IL-13Rα1, whilst yellow arrows show expression of IL-13Rα1 only.

Supplementary Fig S3

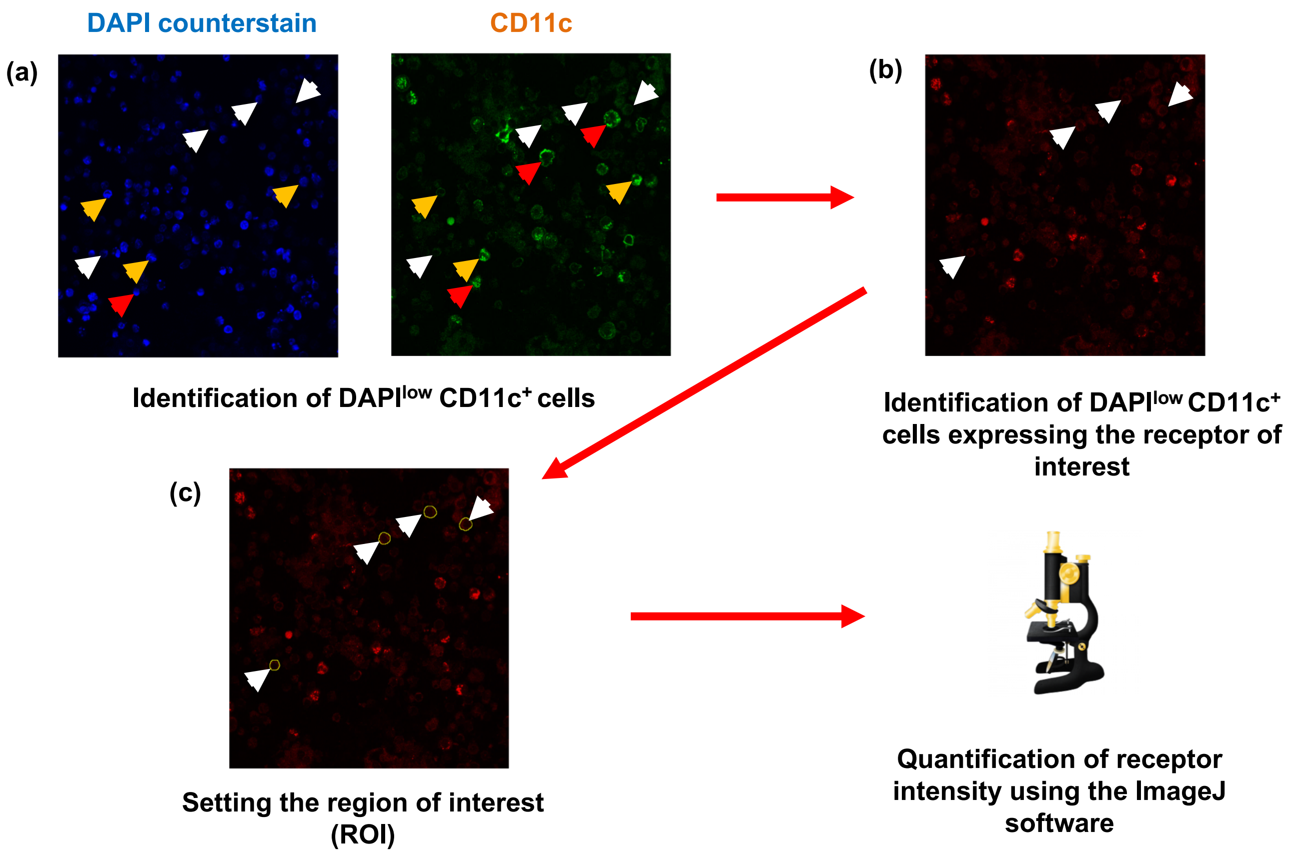

Fig S3. IL-13/ IL-4 receptor intensity quantification strategy using confocal microscopy. (a) White arrows indicate DAPI^{low} CD11c^{+} cells, red arrows and yellow arrows indicate apoptotic cells with unstructured cell membrane morphology and pre-apoptotic cells with high DAPI counterstaining respectively, which were not considered in this study. **(b)** White arrows indicate DAPI^{low} CD11c^{+} cells expressing the receptor of interest, **(c)** each cell of interest was identified by a region of interest (ROI) (yellow circle) (left) and measured the integrated density using the ImageJ software (right). Receptor intensity was calculated as: integrated density of ROI/ area of ROI.

Supplementary Fig S4

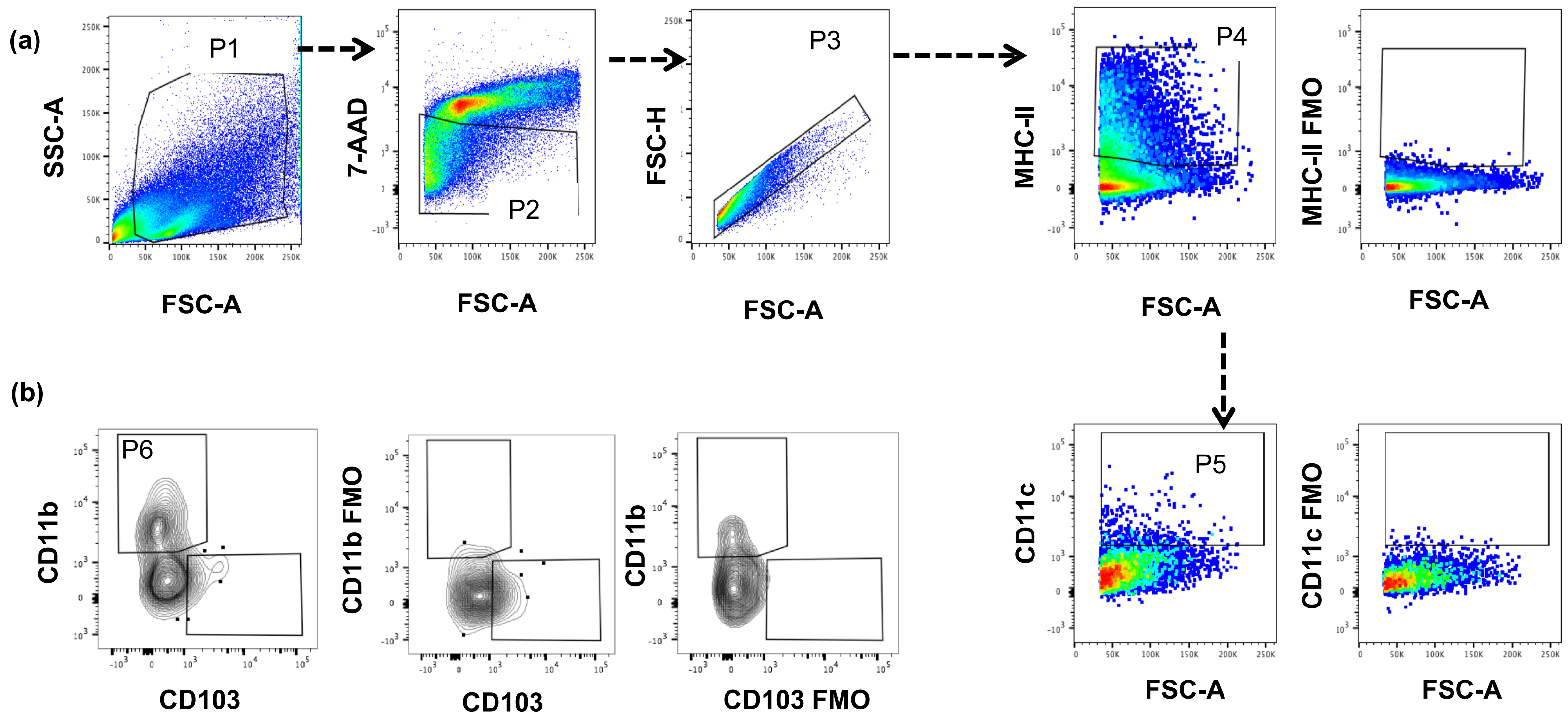

Fig S4. Flow cytometry gating strategy and Fluorescence minus one (FMO) controls used to identify lung cDCs for single cell cell sorting following i.n. viral vector immunisation. Pre-gated cells (P1) were used to gate on 7-AAD$^-$ viable cells (P2), followed by doublet exclusion (P3) based on forward scatter (FSC-H and FSC-A). Cells were then gated on MHC-II-I-Ad$^+$ (P4), followed by total DCs represented as (MHC-II-I-A^{d+} CD11c$^+$ - P5). **(b)** Total lung DCs were further gated on CD11b$^+$ CD103$^-$ cDCs (P6) based on FMO controls as indicated.

Supplementary Fig S5

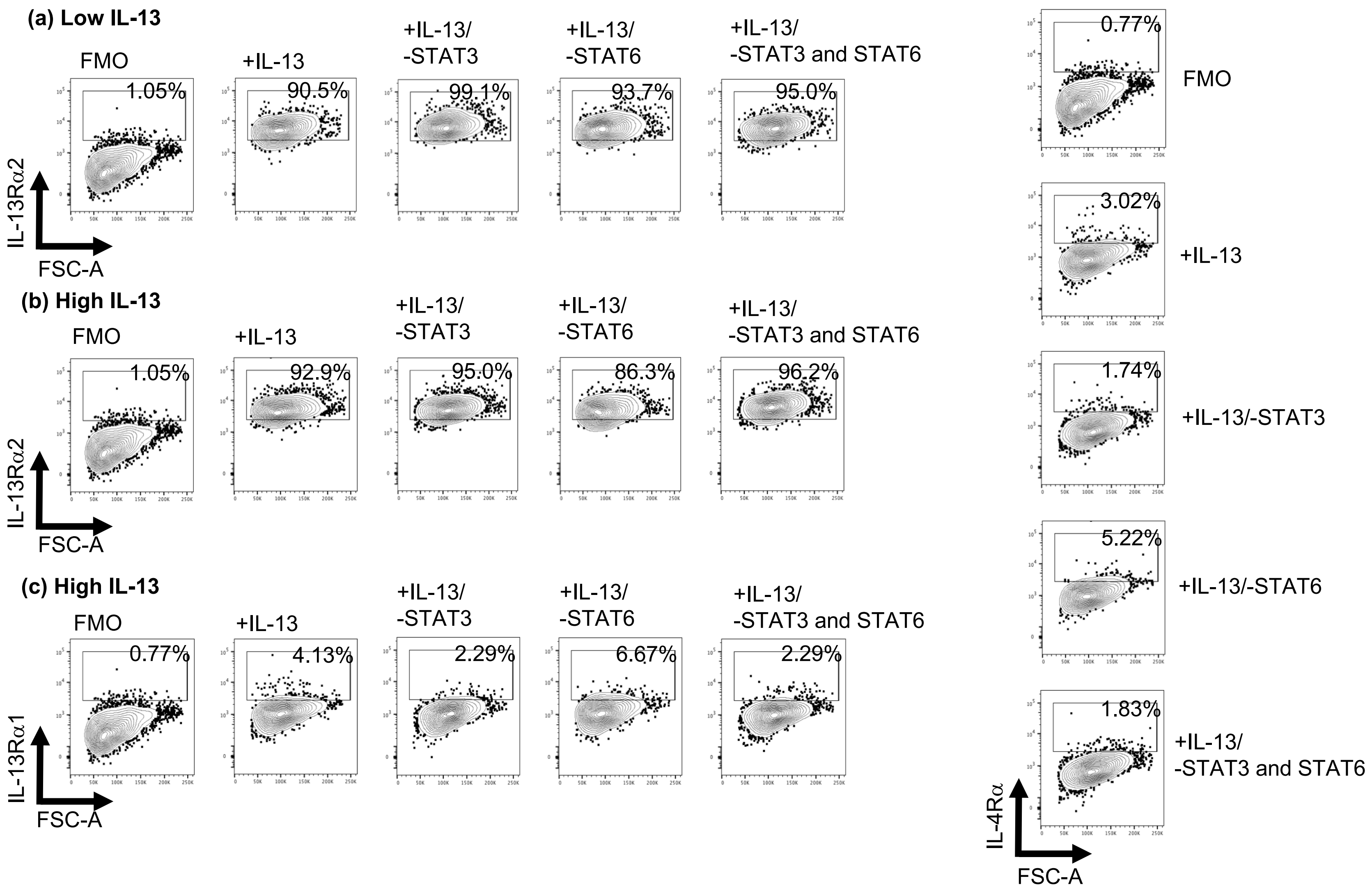

Fig. S5 IL-4 and IL-13 receptor expression following STAT3/ STAT6 inhibition and IL-13 stimulation of lung DCs. Representative FACS plots showing STAT3, STAT6 or STAT3/STAT6 combined inhibitor treated total lung MHC-II-I-A^{d+} CD11c^{+} DCs, further gated on IL-13Rα2 following **(a)** 100pM low IL-13 and **(b)** 10000 pM high IL-13; **(c)** IL-13Rα1 and **(d)** IL-4Rα following 10000 pM high IL-13 stimulation for 3 h.

Supplementary Fig S6

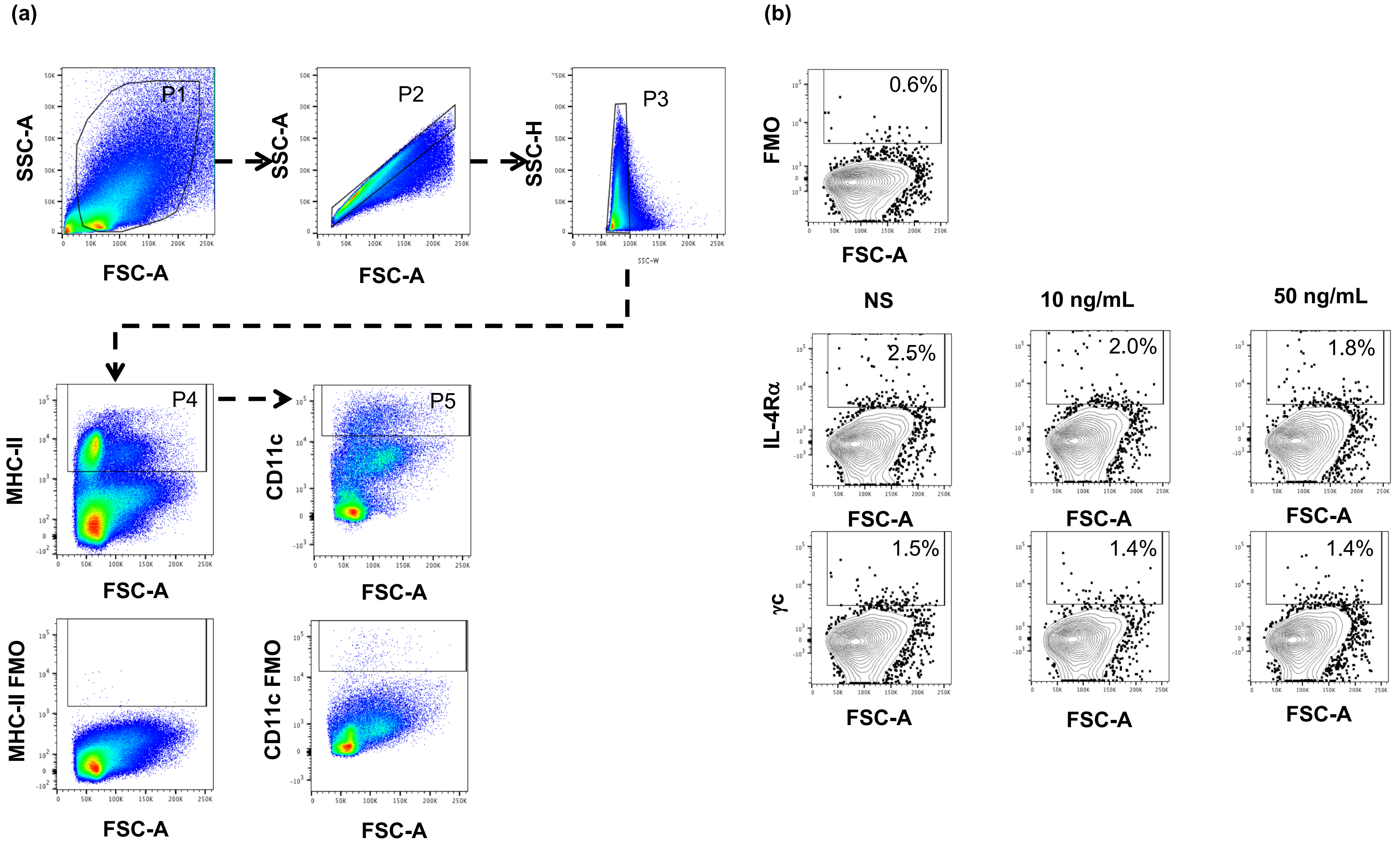

Fig S6. Flow cytometry gating for IL-4 receptors on lung DCs following in vitro IL-4 stimulation. **(a)** Plots show viable cells (P1) followed by gating for single cells based on forward scatter (FSC-H and FSC-A; P2) and side scatter (SSC-H and SSC-W; P3). MHC-II$^+$ (P4) and CD11c$^+$ cells were determined by comparison with FMO controls. Total DCs (MHC-II$^+$ CD11c$^+$ - P5) were gated followed by receptor positive cells, based on appropriate FMO controls. **(b)** Representative flow cytometry plots show IL-4Rα and γc expression following 0.5 h of stimulation with varying concentration of IL-4 (NS, 10 ng/mL and 50 ng/mL) .

Supplementary Fig S7

(a)

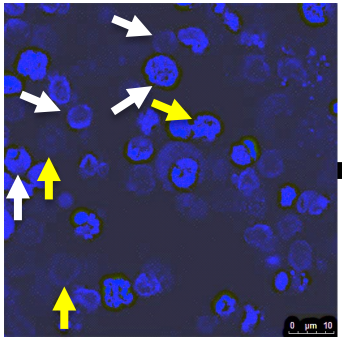

Identification of morphologically viable DAPI^{lo} lung cells

(b)

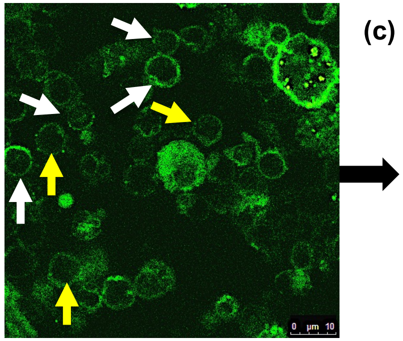

Identification of CD11c$^+$ lung DCs

(c)

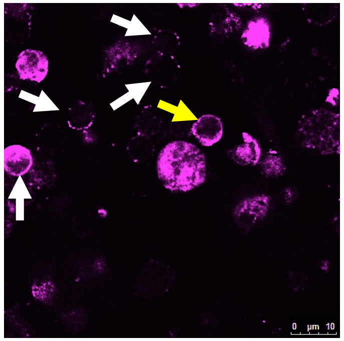

Identification of CD11c$^+$ IL-13Rα2$^+$ lung DCs

(d)

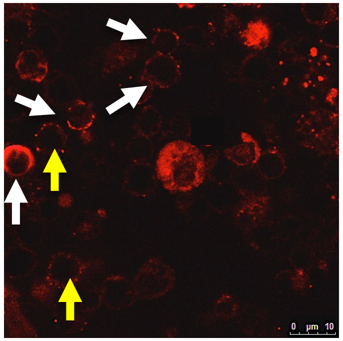

Identification of CD11c$^+$ IFN-γR$^+$ lung DCs

(e)

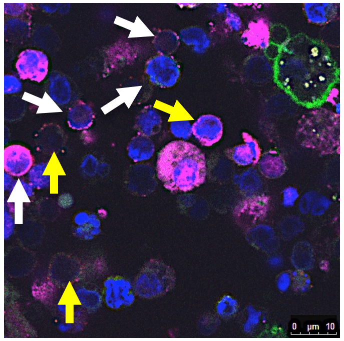

Identification of CD11c$^+$ double positive for IL-13Rα2 and IFN-γR lung DCs

(f)

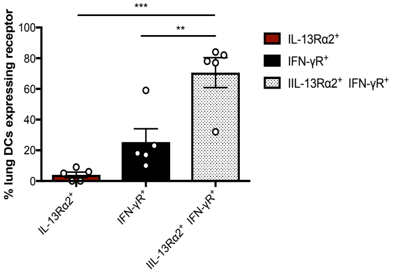

Calculation of proportion of CD11c$^+$ IL-13Rα2$^+$ IFN-γR$^+$ as a percentage of total viable lung DCs

Fig S7. IL-13Rα2 and IFN-γR co-expression and quantification strategy using confocal microscopy. (a) DAPI^{low} cells were identified as viable lung cells followed by **(b)** CD11c$^+$ lung DCs, **(c)** IL-13Rα2$^+$ lung DCs, **(d)** IFN-γR$^+$ lung DCs and **(e)** cells double positive for IL-13Rα2 and IFN-γR. Yellow arrows indicate DAPI^{low} CD11c$^+$ lung DCs expressing a single receptor and and white arrows indicate co-expression of both receptors. **(f)** Bar graph represents percentage of IL-13Rα2$^+$, IFN-γR$^+$ and IL-13Rα2$^+$ IFN-γR$^+$ lung CD11c$^+$ DCs calculated as a proportion of the total viable DAPI^{low} CD11c$^+$ DCs from 5 imaged areas.

Supplementary Fig S8

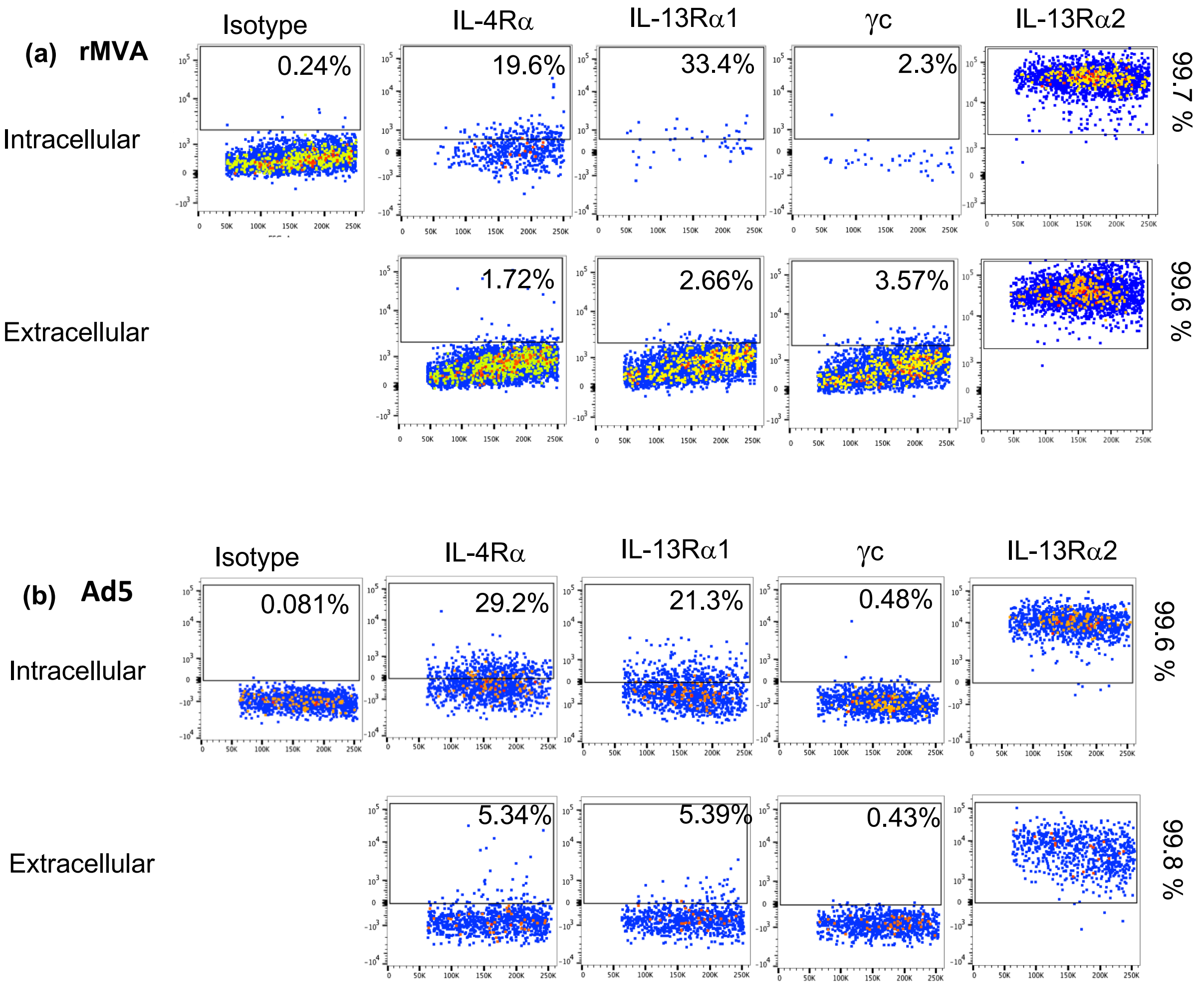

Fig S8. Flow cytometry plots showing IL-4 and IL-13 receptor expression on lung cDCs 24 h post i.n. viral vector vaccination. Representative FACS plots showing intracellular and extracellular expressions of IL-4Rα, IL-13Rα1, γC and IL-13Rα2 on lung CD11b^{+} CD103^{-} cDCs, following 24 h post i.n. vaccination of BALB/c (n=5 per group) with **(a)** rMVA **(b)** Ad5.

Supplementary Fig S9

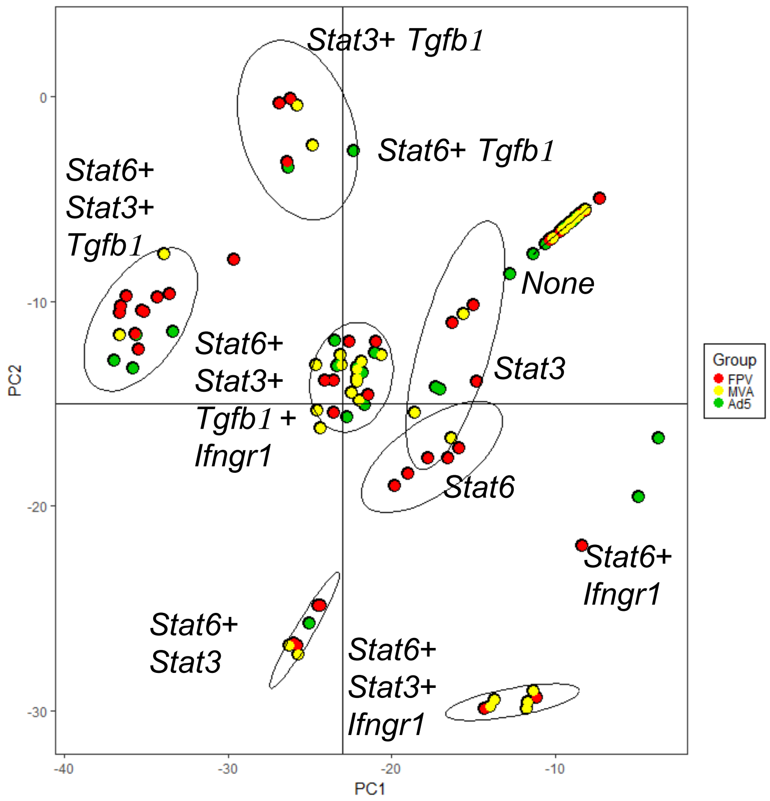

Fig S9. Evaluation of viral vector dependent *Stat3*, *Stat6*, *Tgfb1* and *Ifngr1* expression using PCA and K-means clustering. BALB/c mice (n=3 per group) were vaccinated with rFPV, rMVA or Ad5. 24 h post vaccination sinlge cDCs were sorted from lung suspensions and Fluidigm 48.48 Biomark assay was performed as described in methods. Data indicate the different *Stat3, Stat6, Tgfb1* and *Ifngr1* gene co-expression profiles in cDCs relevant to each viral vector analysed using PCA and K-means clustering as described in the methods. Each point in the K-mean cluster analysis represents a single cell expressing genes within a cluster. These experiments were performed with 48 cDCs per vaccine group.
